# Supplementary material for: Signatures of T cell immunity revealed using sequence similarity with TCRDivER algorithm
Source: Commun Biol. 2023 Mar 31;6:357. doi: 10.1038/s42003-023-04702-8 (PMC10066310; doi:10.1038/s42003-023-04702-8)
Supplement: Supplementary file 2 — Supplementary Information [file 42003_2023_4702_MOESM2_ESM.pdf]

Supplementary Information: Signatures of T cell immunity revealed using sequence similarity with TCRDivER algorithm

Milena Vujović Paolo Marcatili Benny Chain Joseph Kaplinsky
Thomas Lars Andresen

Supplementary Note 1: TCRDivER and Diversity Features

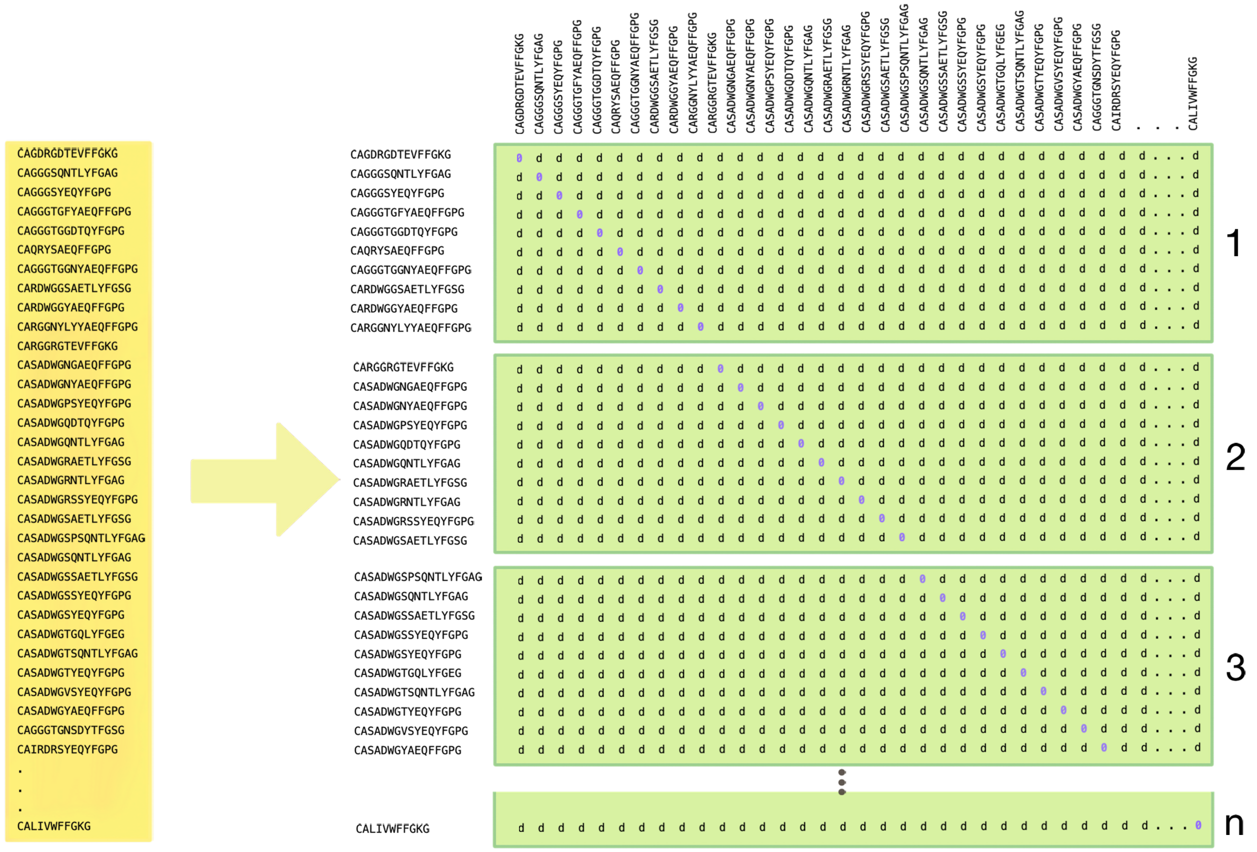

Supplementary Figure 1: An overview of the calculation of the distance matrix. The list of CDR3 sequences is divided into lists of equal length, here 10 sequences, the default value is 100. These 10 CDR3s are then pairwise compared with all the other CDR3s in the total list of CDR3 sequences. Each portion of the distance matrix i.e. chunk has 10 rows and S columns, where S corresponds to the total number of CDR3 clone sequences. In the end there are n chunks, where n is equal to the floored division of total number of sequences by the length of chunk $n = \frac{S}{length\ of\ chunk}$. Distances d are $d(CDR3_i, CDR3_j)$ calculated based on the BLOSUM45 alignment score. The diagonal of the combined distance matrix is 0.

Supplementary Note 1.1: Evaluation of naive diversity of the first order

We start with:

$$\ln\left(D(q)\right) = \ln\left(\sum_{i=1}^{S} p_i^q\right)^{\frac{1}{(1-q)}} \tag{1}$$

Exploring the limit as q approaches 1 allows us to apply L'Hopitals rule:

$$\lim_{q\to1} \ln\left(D(q)\right) = \lim_{q\to1} \frac{\ln\sum_{i=1}^{S} p_i^q}{1-q} = \lim_{q\to1} \frac{\left(\ln\sum_{i=1}^{S} p_i^q\right)'}{(1-q)'} \tag{2}$$

The solution is:

$$\ln\left(D(1)\right) = -\sum_{i=1}^{S} p_i \ln p_i \tag{3}$$

This is equivalent to:

$$D(1) = \frac{1}{p_1^{p_1} p_2^{p_2} \cdots p_i^{p_i}} \tag{4}$$

Supplementary Note 1.2: Evaluating the slope at $q = 1$

We start with the definition of ${}^qD$:

$$D(q) = \left(\sum_{i=1}^{N} p_i^q\right)^{\frac{1}{1-q}} \tag{5}$$

define S as the internal sum

$$S = \sum_{i=1}^{N} p_i^q \tag{6}$$

Now we can write

$$\ln(D(q)) = \frac{1}{1-q} \ln S \tag{7}$$

Let $'$ denote differentiation with respect to q. Now evaluate

$$\ln(D(q))' = \frac{1}{(1-q)^2} \ln S + \frac{1}{1-q} (\ln S)' \tag{8}$$

$$\ln(D(q))' = \frac{\ln S + (1-q)(\ln S)'}{(1-q)^2} \tag{9}$$

To evaluate the limit as q goes to 1 we need to apply l'hopital's rule twice. Calling the numerator t

$$t = \ln S + (1-q)(\ln S)' \tag{10}$$

$$t' = (1-q)(\ln S)'' \tag{11}$$

$$t'' = (1-q)(\ln S)''' - (\ln S)'' \tag{12}$$

Since $\ln S$ and all its derivatives are finite as q goes to 1

$$\lim_{q\to1} t'' = -\lim_{q\to1} (\ln S)'' \tag{13}$$

Call the denominator b

$$b'' = 2 \tag{14}$$

and

$$\lim_{q\to 1} b'' = 2 \tag{15}$$

Putting this together we have

$$\lim_{q\to 1} \ln(D(q))' = \frac{\lim_{q\to 1} t''}{\lim_{q\to 1} b''} \tag{16}$$

$$\lim_{q\to 1} \ln(D(q))' = -\frac{1}{2}(\ln S)''\Big|_{q=1} \tag{17}$$

We need

$$(\ln S)' = \frac{\sum_{i=1}^{N} p_i^q \ln p_i}{S} \tag{18}$$

$$(\ln S)'' = \frac{\sum_{i=1}^{N} p_i^q (\ln p_i)^2}{S} - \frac{(\sum_{i=1}^{N} p_i^q \ln p_i)^2}{S^2} \tag{19}$$

Because p_i is a probability distribution

$$\lim_{q\to 1} S = 1 \tag{20}$$

which means (since the limit of quotient is the quotient of the limits)

$$\lim_{q\to 1} S'' = \sum_{i=1}^{N} p_i^q (\ln p_i)^2 - (\sum_{i=1}^{N} p_i^q \ln p_i)^2 \tag{21}$$

Finally, we want to evaluate $D(q)'$ and then take the limit as q goes to 1.

$$D(q)' = D(q)\,(\ln(D(q)))' \tag{22}$$

and

$$\lim_{q\to 1} D(q)' = \lim_{q\to 1} D(q)\ \lim_{q\to 1} -\frac{1}{2}(\ln S)'' \tag{23}$$

$$\lim_{q\to 1} D(q)' = -\frac{1}{2}\,D(1)\,\left(\sum_{i=1}^{N} p_i^q (\ln p_i)^2 - (\sum_{i=1}^{N} p_i^q \ln p_i)^2\right) \tag{24}$$

To generalise to the case $\mathbf{Z} \neq \mathbf{I}$ we simply have to replace S with

$$S = \sum_{i=1}^{N} p_i (\mathbf{Zp})_i^{q-1}. \tag{25}$$

In this case

$$(\ln S)' = \frac{\sum_{i=1}^{N} p_i (\mathbf{Zp})_i^{q-1} \ln(\mathbf{Zp})_i}{S} \tag{26}$$

$$(\ln S)'' = \frac{\sum_{i=1}^{N} p_i (\mathbf{Zp})_i^{q-1} (\ln \mathbf{Zp})_i)^2}{S} - \frac{(\sum_{i=1}^{N} p_i (\mathbf{Zp})_i^{q-1} \ln(\mathbf{Zp})_i)^2}{S^2} \tag{27}$$

Supplementary Note 1.3: Evaluation of similarity scaled diversity of the first order

We start with:

$$\ln\ D(q,\lambda) = \ln\left(\sum_{i=1}^{S} p_i (\mathbf{Zp})_i^{q-1}\right)^{\frac{1}{(1-q)}} \tag{28}$$

Rewriting the equation, calculating the limit as $q \to 1$ and applying L'Hopitals rule:

$$\lim_{q\to 1} \ln\ D(q,\lambda) = \lim_{q\to 1} \frac{\ln \sum_{i=1}^{S} p_i (\mathbf{Zp})_i^{q-1}}{1-q} = \lim_{q\to 1} \frac{\left(\ln \sum_{i=1}^{S} p_i (\mathbf{Zp})_i^{q-1}\right)'}{(1-q)'} \tag{29}$$

The result is:

$$\ln\left(D(1,\lambda)\right) = -\sum_{i=1}^{S} p_i \ln\left(\mathbf{Zp}\right)_i \tag{30}$$

, which is equivalent to:

$$D(q,\lambda) = \frac{1}{(\mathbf{Zp})_1^{p_1}(\mathbf{Zp})_2^{p_2}\cdots(\mathbf{Zp})_i^{p_i}} \tag{31}$$

We would like to note that the use of L'Hopitals rule has been established in literature to link scaled diversity measueres to Shannon entropy [40].

Supplementary Note 1.4: Evaluation of naive diversity of the infinity order

We start with the formula for naive diversity and extract the largest clone frequency p_{max}:

$$D(q) = \big(\sum_{i=1}^{S} p_i^q\big)^{\frac{1}{1-q}} = (p_{max})^{\frac{q}{1-q}}\big(1+\sum_{j=1}^{S} p_j^{'q}\big)^{\frac{1}{1-q}} \tag{32}$$

,where $p_j^{'} = \frac{p_j}{p_{max}}$ for $j \neq max$, and p_{max} is represented in the first term of the sum. Since a limit of products is a product of limits, it follows:

$$\lim_{q\to\infty} D(q) = \lim_{q\to\infty}(p_{max})^{\frac{q}{1-q}} \lim_{q\to\infty}(1+\sum_{j=1}^{S} p_j^{'q})^{\frac{1}{1-q}} \tag{33}$$

The first limit is evaluated as:

$$\lim_{q\to\infty}(p_{max})^{\frac{q}{1-q}} = \frac{1}{p_{max}} \tag{34}$$

The second limit is evaluated by taking the logarithm:

$$\log\left(\lim_{q\to\infty}(1+\sum_{j=1}^{S} p_j^{'q})^{\frac{1}{1-q}}\right) = \lim_{q\to\infty}\log\left((1+\sum_{j=1}^{S} p_j^{'q})^{\frac{1}{1-q}}\right) = \lim_{q\to\infty}\frac{1}{(1-q)}\log(1+\sum_{j=1}^{S} p_j^{'q}) \tag{35}$$

Since $0 < \sum_j^S p_j^{'q} < 1$, the bounds of logarithm are:

$$0 < \log(1+\sum_{j=1}^{S} p_j^{'q}) < \log 2 \tag{36}$$

, which gives:

$$\lim_{q\to\infty}\frac{1}{(1-q)}\log(1+\sum_{j=1}^{S} p_j^{'q}) = 0 \tag{37}$$

$$\implies \log\left(\lim_{q\to\infty}(1+\sum_{j=1}^{S} p_j^{'q})^{\frac{1}{1-q}}\right) = 0 \tag{38}$$

$$\implies \lim_{q\to\infty}(1+\sum_{j=1}^{S} p_j^{'q})^{\frac{1}{1-q}} = 1 \tag{39}$$

$$\implies \lim_{q\to\infty} D(q) = \frac{1}{p_{max}} \tag{40}$$

Supplementary Note 1.5: Evaluation of similarity scaled diversity of the infinity order

We start with:

$$D(q,\lambda) = \Big(\sum_{i=1}^{S} p_i(\mathbf{Zp})_i^{q-1}\Big)^{\frac{1}{(1-q)}} = ((\mathbf{Zp})_{max})^{\frac{q-1}{1-q}}\Big(p_{max}(1+\sum_j p_j^{'}(\mathbf{Zp})_j^{'q-1}\Big)^{\frac{1}{1-q}} \tag{41}$$

where the term that has been pulled out is the one for which $(\mathbf{Zp})_i$ is maximum. The p_{max} is the corresponding p_i. As before, the $p_j^{'}$ are defined as $\frac{p_j}{p_{max}}$ for $j \neq max$ and $(\mathbf{Zp})_j^{'}$ is defined as $\frac{(\mathbf{Zp})_j}{(\mathbf{Zp})_{max}}$. Again, the limit splits in to two factors:

$$\lim_{q\to\infty}(\mathbf{Zp})_{max})^{\frac{q-1}{1-q}} = \frac{1}{(\mathbf{Zp})_{max})} \tag{42}$$

Taking the log of the second term gives:

$$\lim_{x\to\infty}\frac{1}{1-q}\log\Big(p_{max}(1+\sum_j p_j^{'}(\mathbf{Zp})_j^{'q-1}\Big) \tag{43}$$

and now the log is bounded by:

$$\log p_{max} < \log\Big(p_{max}(1+\sum_j p_j^{'}(\mathbf{Zp})_j^{'q-1}\Big) < \log 2 \tag{44}$$

so again the limit of the log second factor in (*) is 0, and limit of the factor itself is 1. The end result is:

$$\lim_{q\to\infty} D(q,\lambda) = \frac{1}{(\mathbf{Zp})_{max}} \tag{45}$$

which reduces to the correct limit when **Z**=**I** which is the naive diversity.

Supplementary Note 1.6: Evaluation $\Delta ln(D(q,\lambda))$ for small λ: Perturbation around $\lambda = 0$

Conjecture: gradient of $\frac{D(q,\lambda)}{dq}\Big|_{q=0}$ is a decreasing function of λ. N.B. $D(q,\lambda)$ is an increasing function of λ for all q.

We start with the assumption that for λ around 0:

$$\ln(D(q,\lambda)) \propto \lambda \tag{46}$$

Where $\ln(D(q,\lambda))$ is:

$$\ln(D(q,\lambda)) = \ln\Big(\sum_{i=1}^{S} p_i(\mathbf{Zp})_i^{q-1}\Big)^{\frac{1}{(1-q)}} \tag{47}$$

$$\ln(D(q,\lambda)) = \frac{1}{1-q}\ln\Big(\sum_{i=1}^{S} p_i(\mathbf{Zp})_i^{q-1}\Big) \tag{48}$$

$$\ln(D(q,\lambda)) = \frac{1}{1-q}\ln\Big(\sum_{i=1}^{S} p_i\big(\sum_{j=1}^{S} p_j e^{-\lambda d_{ij}}\big)^{q-1}\Big) \tag{49}$$

For $\lambda \to 0$ by applying Taylor expansion $e^{-\lambda d_{ij}}$ reduces to $1-\lambda d_{ij}$ which gives:

$$\ln(D(q,\lambda)) \approx \frac{1}{1-q}\ln\Big(\sum_{i=1}^{S} p_i\big(\sum_{j=1}^{S} p_j(1-\lambda d_{ij})\big)^{q-1}\Big) \tag{50}$$

We can then rewrite:

$$\big(\sum_{j=1}^{S} p_j(1-\lambda d_{ij})\big)^{q-1} \approx \Big(p_0(1-\lambda d_{i0}) + p_1(1-\lambda d_{i1}) + \cdots + p_j(1-\lambda d_{ij})\Big)^{q-1} \tag{51}$$

$$\big(\sum_{j=1}^{S} p_j(1-\lambda d_{ij})\big)^{q-1} \approx \Big(1-\lambda\big(\sum_{j=1}^{S} p_j d_{ij}\big)\Big)^{q-1} \tag{52}$$

By applying the binomial expansion we arrive at:

$$\big(\sum_{j=1}^{S} p_j(1-\lambda d_{ij})\big)^{q-1} \approx \Big(1-(q-1)\lambda\big(\sum_{j=1}^{S} p_j d_{ij}\big)\Big) \tag{53}$$

By substituting the derived expressions in the formula for $\ln(D(q,\lambda))$ and keeping in mind that $\sum_{i=1}^{S} p_i = 1$, we can write:

$$\ln(D(q,\lambda)) = \frac{1}{1-q}\ln\Big(\sum_{i=1}^{S} p_i\big(\sum_{j=1}^{S} p_j e^{-\lambda d_{ij}}\big)^{q-1}\Big) \tag{54}$$

$$\ln(D(q,\lambda)) \approx \frac{1}{1-q}\ln\Big(\sum_{i=1}^{S} p_i\big(1-(q-1)\lambda\big(\sum_{j=1}^{S} p_j d_{ij}\big)\big)\Big) \tag{55}$$

$$\ln(D(q,\lambda)) \approx \frac{1}{1-q}\ln\Big(1-\sum_{i=1}^{S} p_i(q-1)\lambda\big(\sum_{j=1}^{S} p_j d_{ij}\big)\Big) \tag{56}$$

$$\ln(D(q,\lambda)) \approx \frac{1}{1-q}\ln\Big(1-(q-1)\lambda\sum_{i=1}^{S} p_i\big(\sum_{j=1}^{S} p_j d_{ij}\big)\Big) \tag{57}$$

By applying the linear approximation $ln(1-x) \approx -x$, we finally arrive:

$$\ln(D(q,\lambda)) \approx \frac{1}{1-q}\Big(-(q-1)\lambda\sum_{i=1}^{S} p_i\big(\sum_{j=1}^{S} p_j d_{ij}\big)\Big) \tag{58}$$

$$\ln(D(q,\lambda)) \approx \lambda\sum_{i=1}^{S} p_i\big(\sum_{j=1}^{S} p_j d_{ij}\big) \tag{59}$$

Note that the final form of the evaluation of $D(q,\lambda)$ for $\lambda \to 0$ is independent of the order of diversity q. It is solely dependent on the distance between CDR3 sequences weighted by their respective frequencies.

Supplementary Note 1.7: Evaluation $\Delta ln(D(q,\lambda))$ for small λ and it's relationship to distance

By evaluating $\Delta ln(D(q,\lambda))$ for two values of small λ, where $\lambda^{'} > \lambda^{''}$ we arrive at:

$$\Delta ln(D(q,\lambda)) \approx D(q,\lambda') - D(q,\lambda)'' \tag{60}$$

$$\Delta ln(D(q,\lambda)) \approx \lambda^{'}\sum_{i=1}^{S} p_i\big(\sum_{j=1}^{S} p_j d_{ij}\big) - \lambda^{'}\sum_{i=1}^{S} p_i\big(\sum_{j=1}^{S} p_j d_{ij}\big) \tag{61}$$

$$\Delta ln(D(q,\lambda)) \approx (\lambda^{'} - \lambda^{''})\sum_{i=1}^{S} p_i\big(\sum_{j=1}^{S} p_j d_{ij}\big) \tag{62}$$

It is evident that $\Delta ln(D(q,\lambda))$ is linearly dependent on the distances between CDR3s and their probabilities. In the case of two hypothetical repertoires, **I** and **II**, which have a uniform distribution of CDR3 frequencies within the repertoire $p_i^I = p_i^{II} = p$ and distances between CDR3s $d_{ij}^I > d_{ij}^{II}$, $\Delta ln(D(q,\lambda))$ for repertoire **I** is larger than $\Delta ln(D(q,\lambda))$ for repertoire **II**. That is with the increase of similarity between CDR3s, the area between the curves for small λs decreases. Alternatively, if the distances between CDR3s of the two repertoires are the same $d_{ij}^I = d_{ij}^{II} = d$, and the distribution is still uniform, but the number of clones differs so that repertoire **I** has less clones than **II** i.e. $p_i^I > p_i^{II}$, then $\Delta ln(D(q,\lambda))$ is larger than $\Delta ln(D(q,\lambda))$. Meaning that repertoires with more abundant clones have a larger $\Delta ln(D(q,\lambda))$ for small λs.

Supplementary Note 1.8: Evaluation $\Delta ln(D(q, \lambda))$ for larger λs and it's relationship to distance

In order to evaluate the relationship between CDR3 clone distance and the area between the curves of larger λs we have constructed three mock repertoires. The repertoires constist of 100 CDR3s that are uniformly distributed in the repertoire, i.e. $p_i = \frac{1}{S} = \frac{1}{100} = 0.01$. For each mock repertoire a mock distance matrix was calculated so that the distance between the CDR3s within the repertoire were equal, but that they differ between the repertoires. The distances were $d^{I}_{i,j} = 0.05$, $d^{II}_{i,j} = 0.1$ and $d^{III}_{i,j} = 0.5$, for repertoire I, II and III respectively when $i \neq j$, else $d_{i,j} = 0$ for $i = j$. Individual λ curves of the diversity profiles straight lines - a remnant of uniform distribution of CDR3 frequencies in the repertoire (Figure 2)

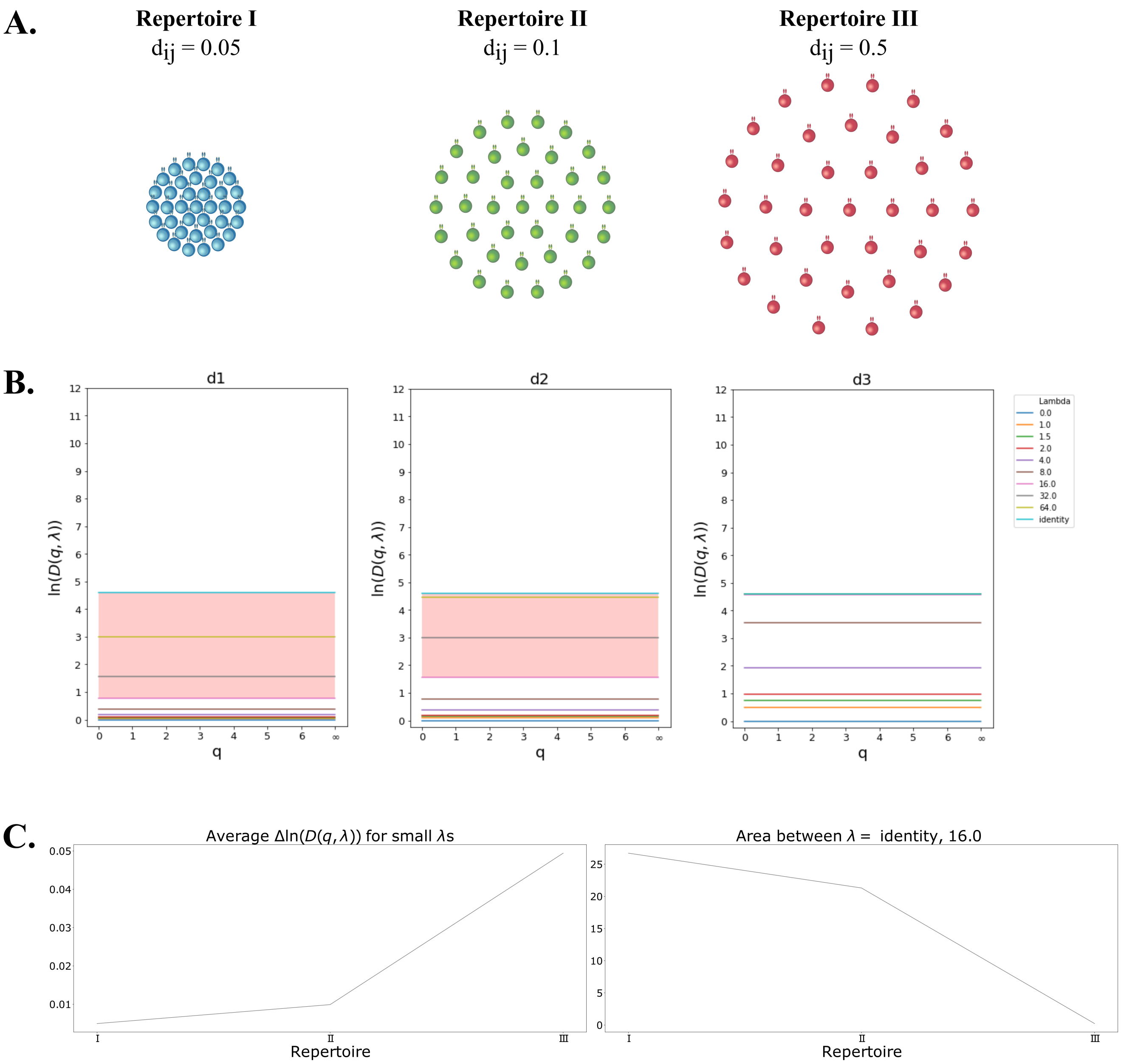

Supplementary Figure 2: Effect of CDR3 distance shown in three mock repertoires with a uniform distribution of 100 CDR3 clones in in the repertoire. **A.** Schematic representation of the three mock repertoires with the distances d_{ij} between CDR3s increasing from repertoire I to III. **B.** Diversity profiles calculated based on the probability distribution and d_{ij} for CDR3s in the mock repertoires. The frequency of seeing each CDR3 clone in all the repertoires, since they consists of 100 uniformly distributed CDR3s, is $p_i = \frac{1}{100} = 0.01$ **C.** Calculated values of average $\Delta \ln(D(q, \lambda))$ for small λs and calculated area between λ identity and 16 curves for the three repertoires, shown left to right respectively.

Supplementary Note 2: Murine Dataset overview, analysis and diversity profiles

Supplementary Table 1: Murine Dataset Subsampling: Overview of number CDR3 clones prior and post subsampling in $CD4^+$ TCR repertoires of the murine dataset

Sample name	Treatment	Sample collection time (days)	Number clones prior to sampling	Number of clones after sampling
SB1_AAA	CFA	5	655436	30400
SB1_CCG	CFA	5	250334	29493
SB1_TTG	CFA	5	1091977	32263
SB1_ACC	CFA	14	411320	27156
SB1_CTA	CFA	14	443454	25445
SB1_GCT	CFA	14	357390	25435
SB2_ATT	CFA	60	252947	19626
SB2_CCG	CFA	60	253711	23146
SB2_CGT	CFA	60	154234	15539
SB1_ATT	CFA+OVA	5	805062	31315
SB1_CAC	CFA+OVA	5	470031	30520
SB1_GTC	CFA+OVA	5	428108	32077
SB1_AGG	CFA+OVA	14	572343	33190
SB1_GAG	CFA+OVA	14	437636	27132
SB1_TAT	CFA+OVA	14	581210	32121
SB2_CAC	CFA+OVA	60	153275	16251
SB2_GCT	CFA+OVA	60	197386	20608
SB2_GTC	CFA+OVA	60	193514	17966
CPX1A_GGA	Non-immunised	0	413430	32295
CPX1A_TTG	Non-immunised	0	283449	30541
CPX1B_CAC	Non-immunised	0	171819	30131
EAE1A_GGA	Non-immunised	0	201127	28170
EAE1A_TTG	Non-immunised	0	155225	28699
EAE1B_CCG	Non-immunised	0	80632	14866
EAE1B_TTG	Non-immunised	0	89643	21506
SB1_TGC	Non-immunised	0	284924	21727

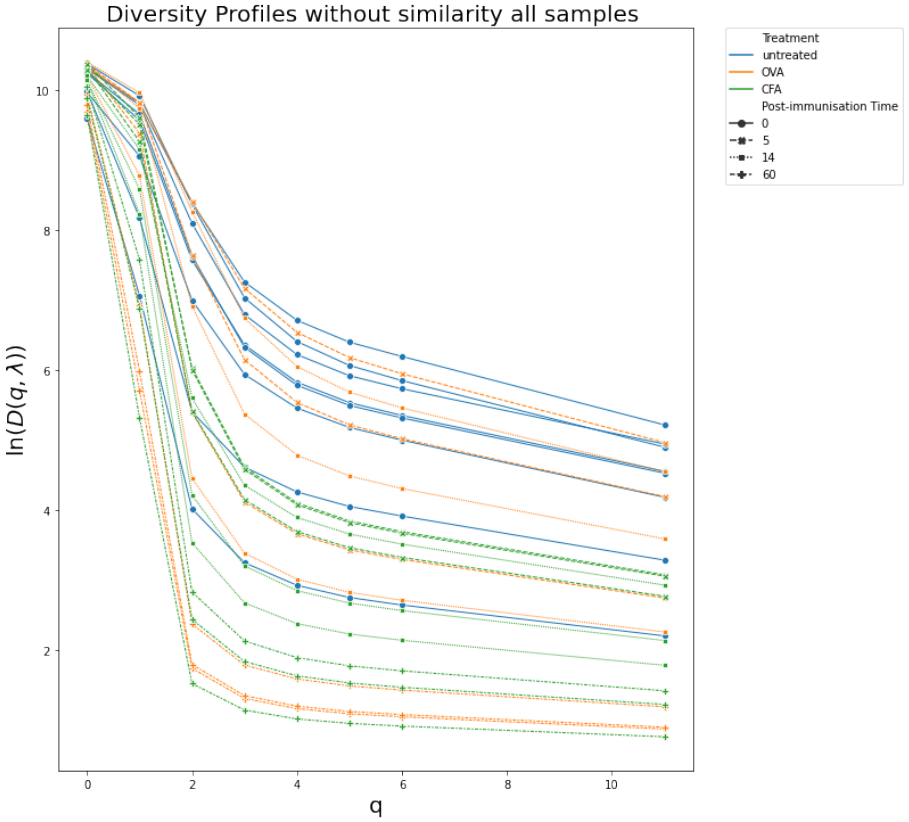

Supplementary Figure 3: Naive ($q = 0$) diversity profiles plotted for all murine samples. Frequent crossings of the curves can be observed illustrating that the rank order of samples depends on the specific choice of index.

Supplementary Note 2.1: Comparison of Simpson and Shannon diversity index for repertoires in the murine dataset

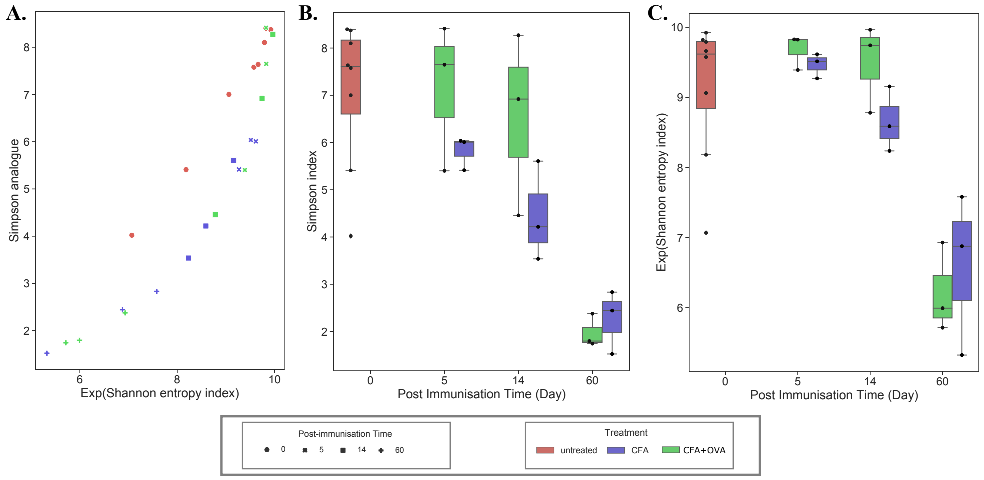

Supplementary Figure 4

Supplementary Note 2.2: Diversity profiles of murine dataset calculated using BLOSUM45 $CDR_{\beta}3$ distances

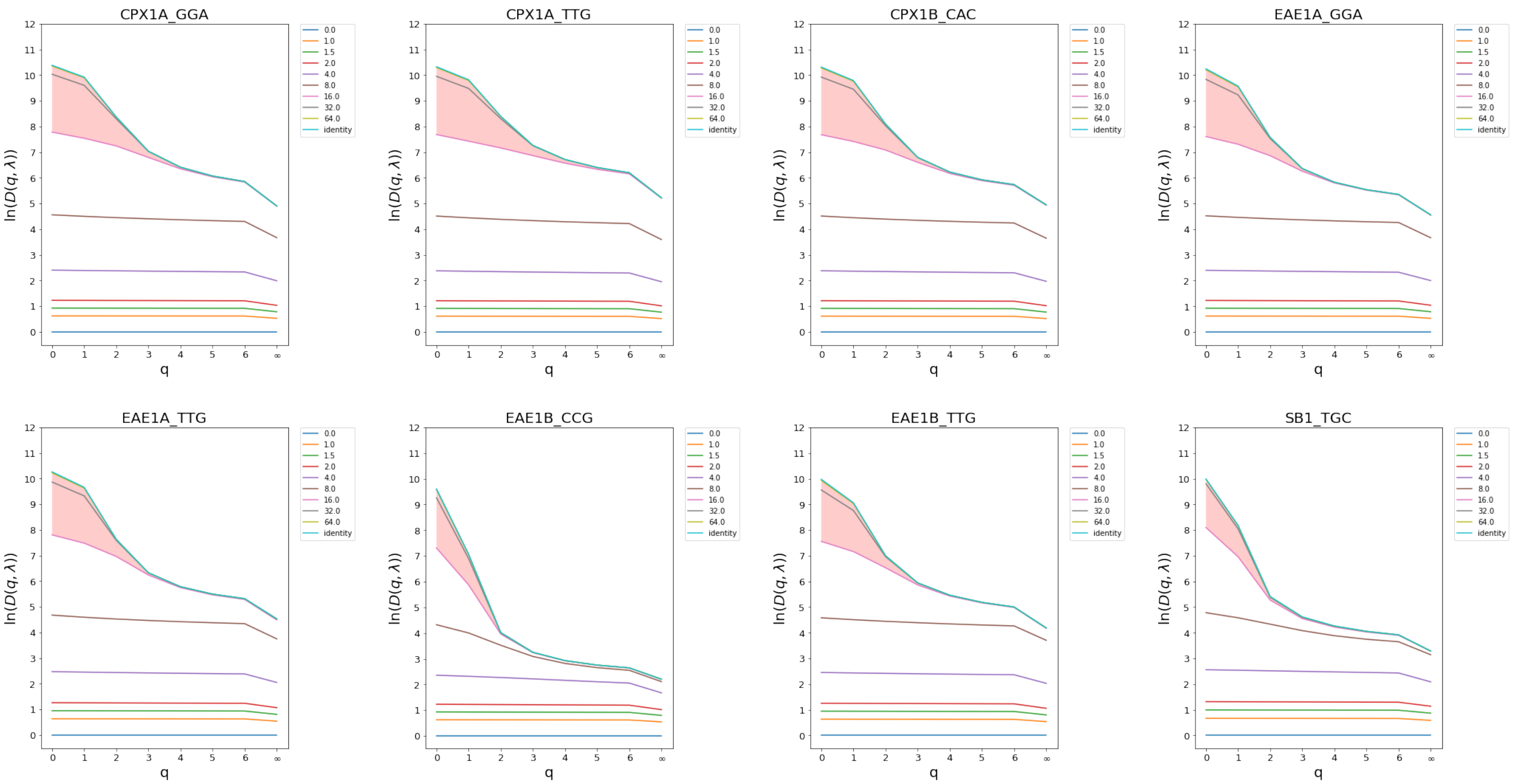

Supplementary Figure 5: Diversity profiles from repertoires of the **Untreated** group in the murine dataset. $CDR_{\beta}3$ distance calculated using BLOSUM45 distance matrix.

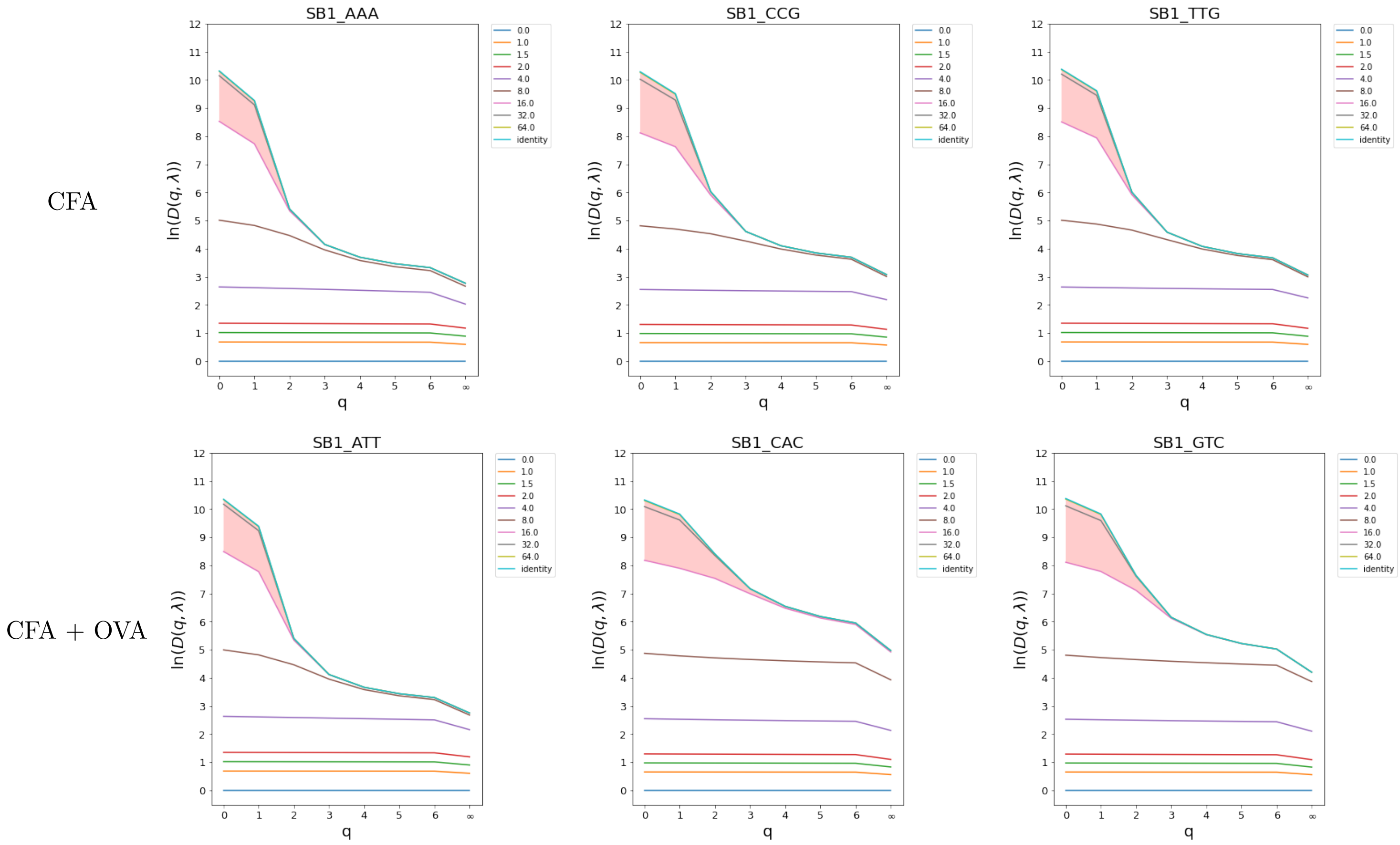

Supplementary Figure 6: Diversity profiles from repertoires of the immunised mice group in the murine dataset collected **5 days** post immunisation. $CDR_\beta 3$ distance calculated using BLOSUM45 distance matrix.

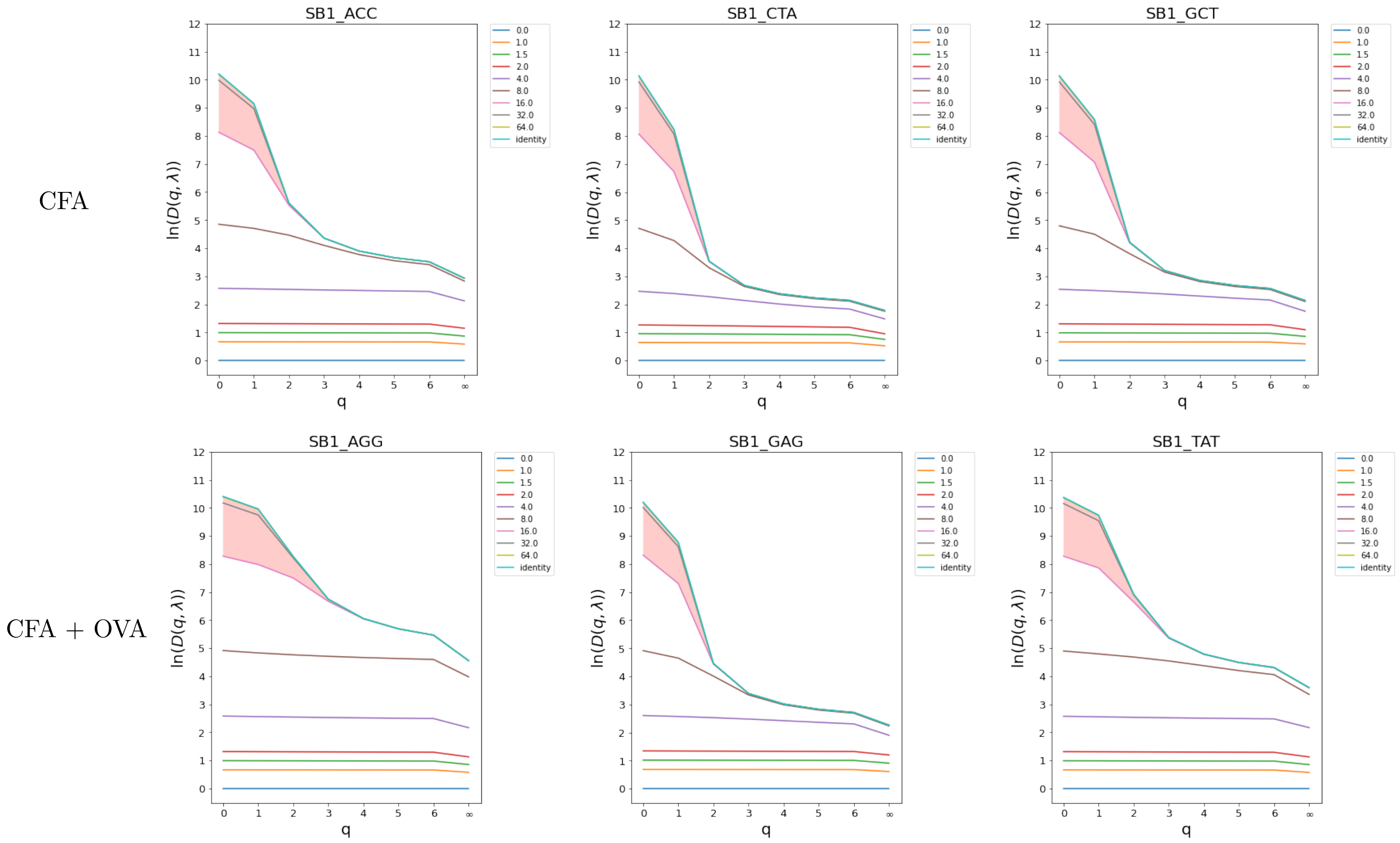

Supplementary Figure 7: Diversity profiles from repertoires of the immunised mice group in the murine dataset collected **14 days** post immunisation. CDR$_\beta$3 distance calculated using BLOSUM45 distance matrix.

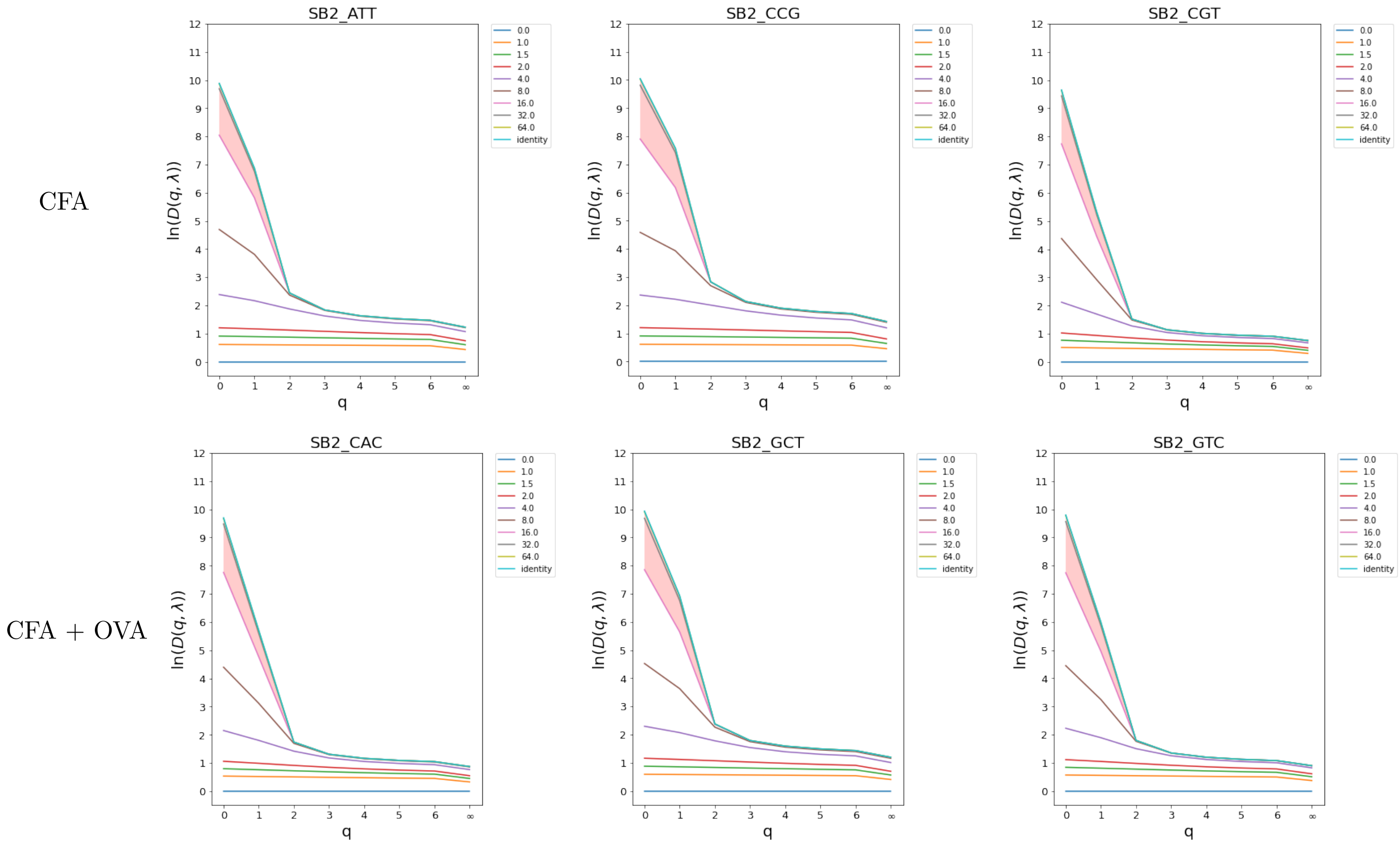

Supplementary Figure 8: Diversity profiles from repertoires of the immunised mice group in the murine dataset collected **60 days** post immunisation. CDR$_{\beta}$3 distance calculated using BLOSUM45 distance matrix.

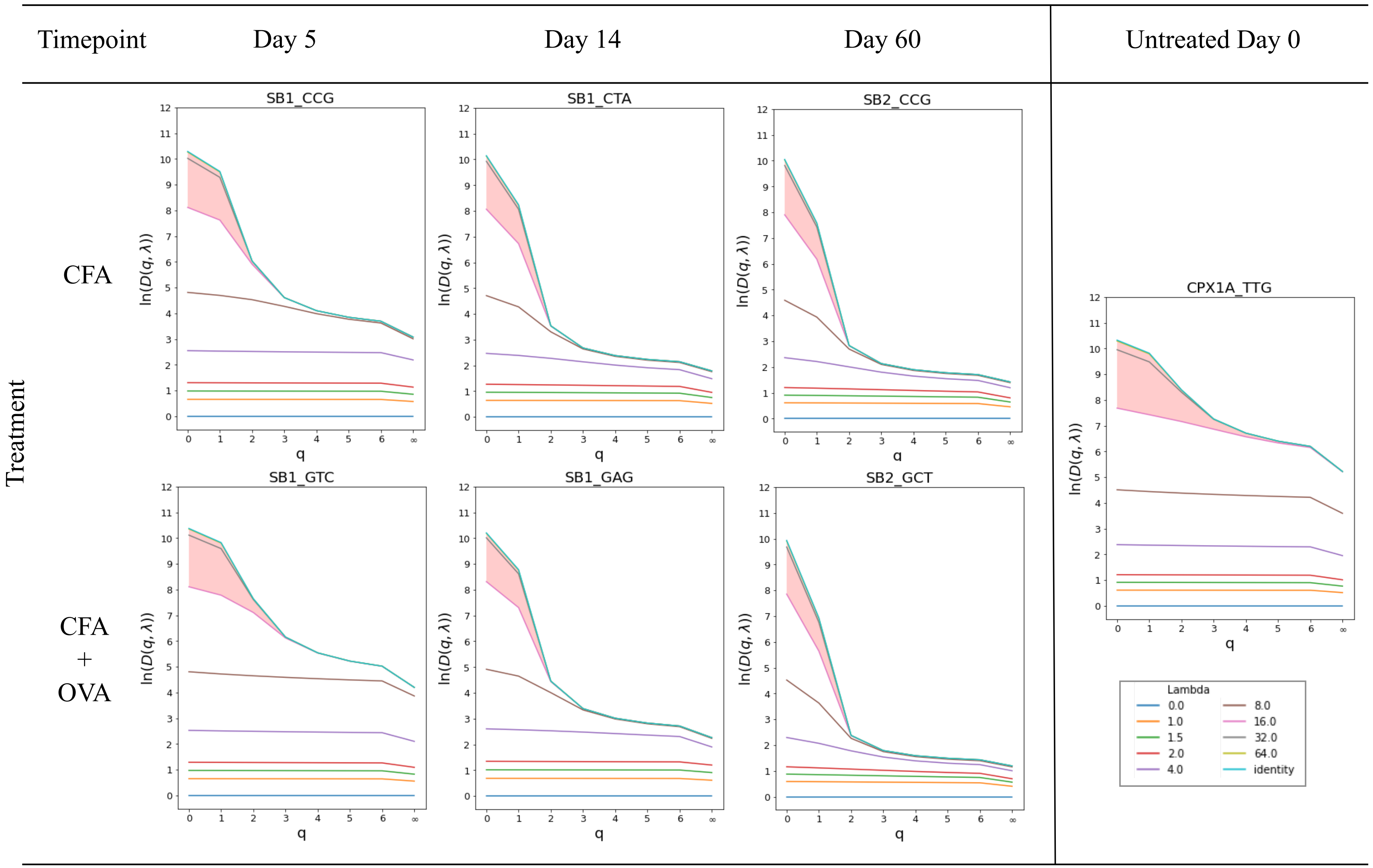

Supplementary Figure 9: Diversity profiles from the repertoires of the murine dataset. Diversity profile of only one sample per group is shown for easy comparison and overview. The rest of the diversity profiles can be found in table 2-5. DivPs of repertoires stemming from immunisation have been shown to the left, while the untreated is shown on the right. The legend for all diversity profiles is shown at the bottom right. The highlighted area represents the area between $q = 16.0$ and identity curves. It highlights the change in repertoire $CDR_{\beta}3$ similarity unification for repertoires of different origin. $CDR_{\beta}3$ distance calculated using BLOSUM45 distance matrix.

Supplementary Note 2.3: PCA on natural logarithm transformed values of diversity with BLOSUM45 distance as CDR3$_\beta$ distance

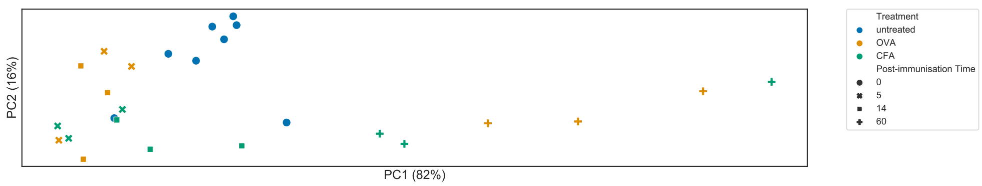

Supplementary Figure 10: Principal Components Analysis on true diversity $D(q, \lambda)$ calculated for the murine dataset using the BLOSUM45 distance for CDR$_\beta$3. The aspect ratio corresponds to variation found by PCA.

Supplementary Note 2.4: Diversity profiles of murine dataset calculated using Atchley factor CDR$_\beta$3 distances

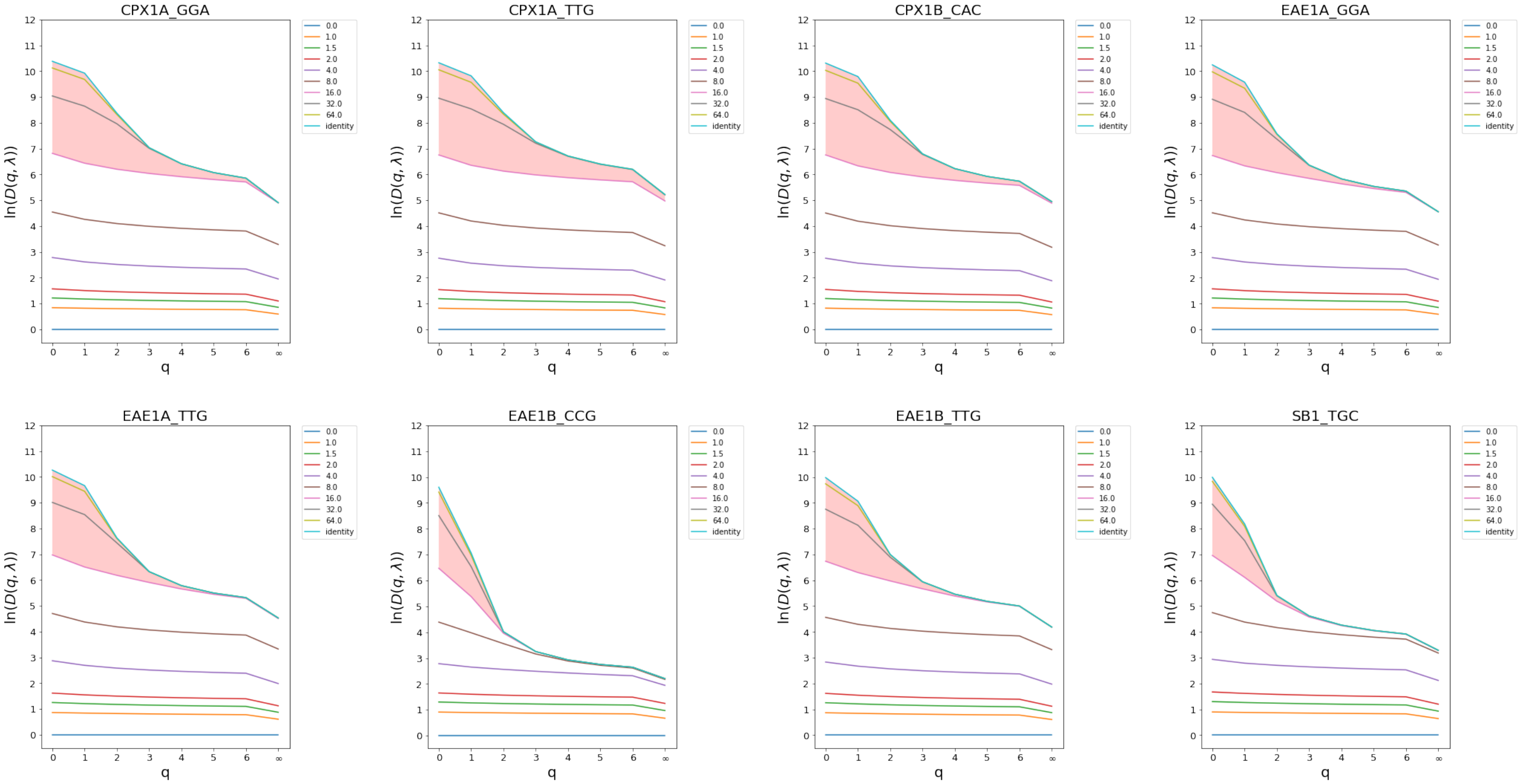

Supplementary Figure 11: Diversity profiles from repertoires of the **Untreated** group in the murine dataset. CDR$_\beta$3 distance calculated using Atchley factor distance.

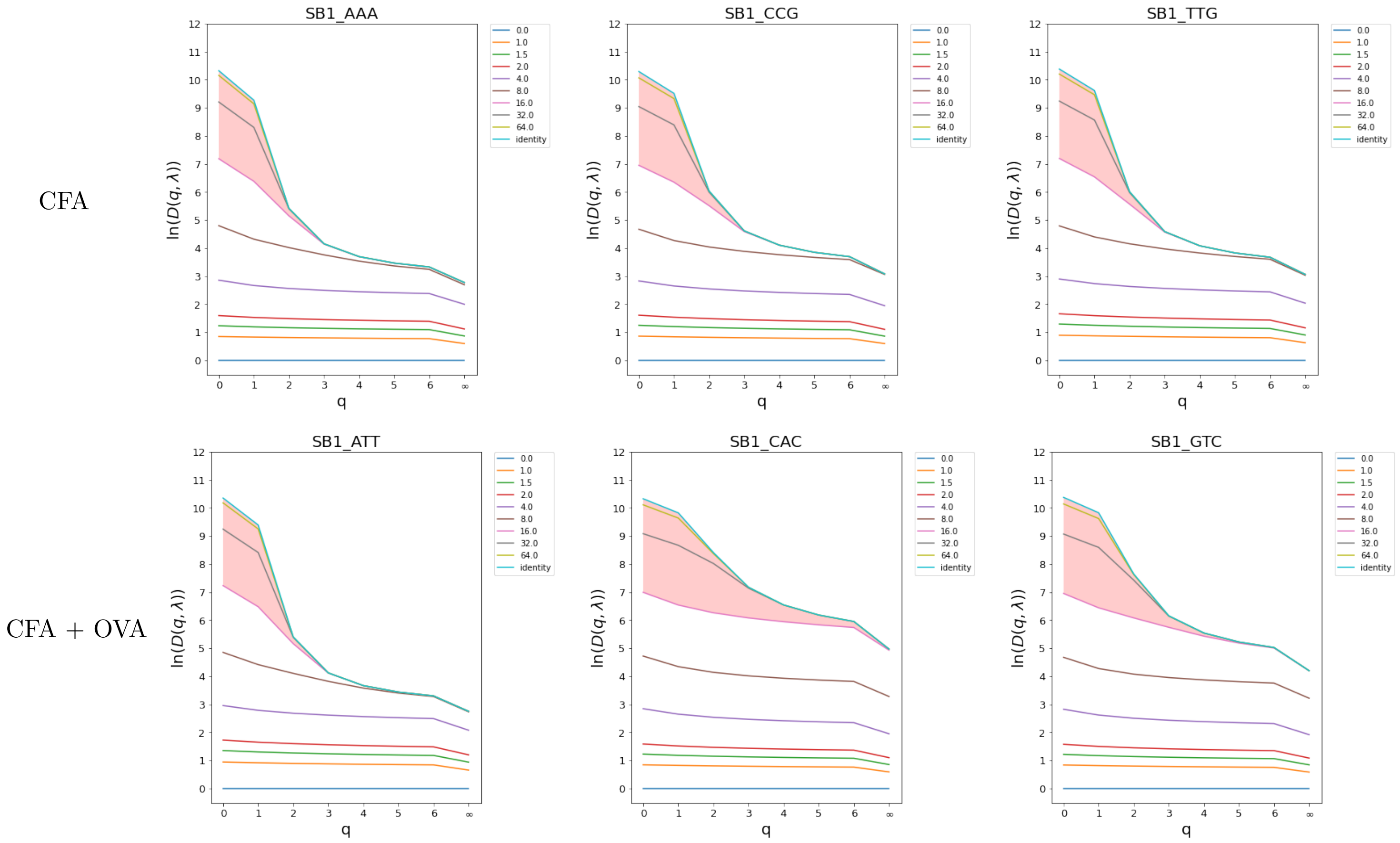

Supplementary Figure 12: Diversity profiles from repertoires of the immunised mice group in the murine dataset collected **5 days** post immunisation. CDR$_\beta$3 distance calculated using Atchley factor distance.

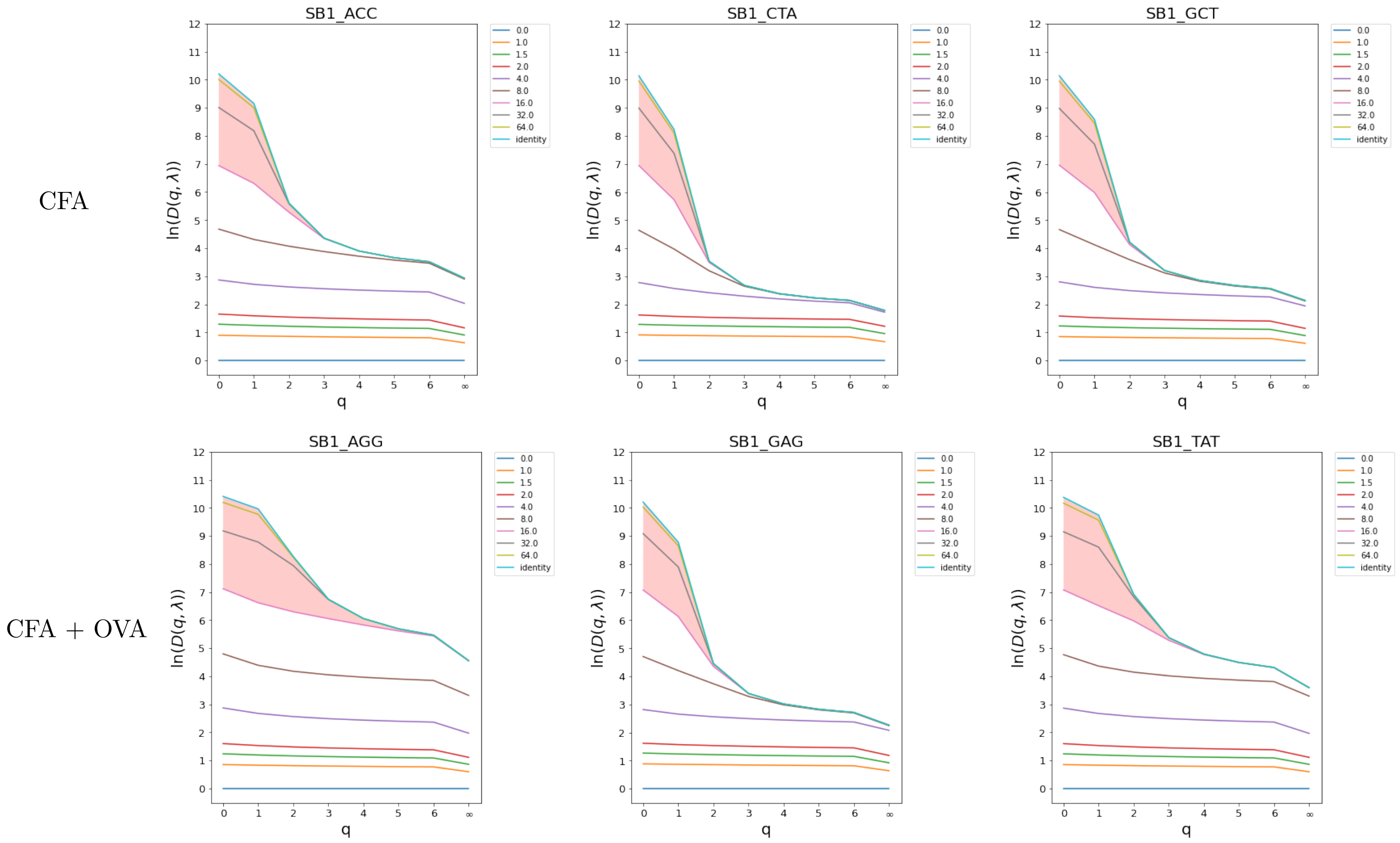

Supplementary Figure 13: Diversity profiles from repertoires of the immunised mice group in the murine dataset collected **14 days** post immunisation. $CDR_{\beta}3$ distance calculated using Atchley factor distance.

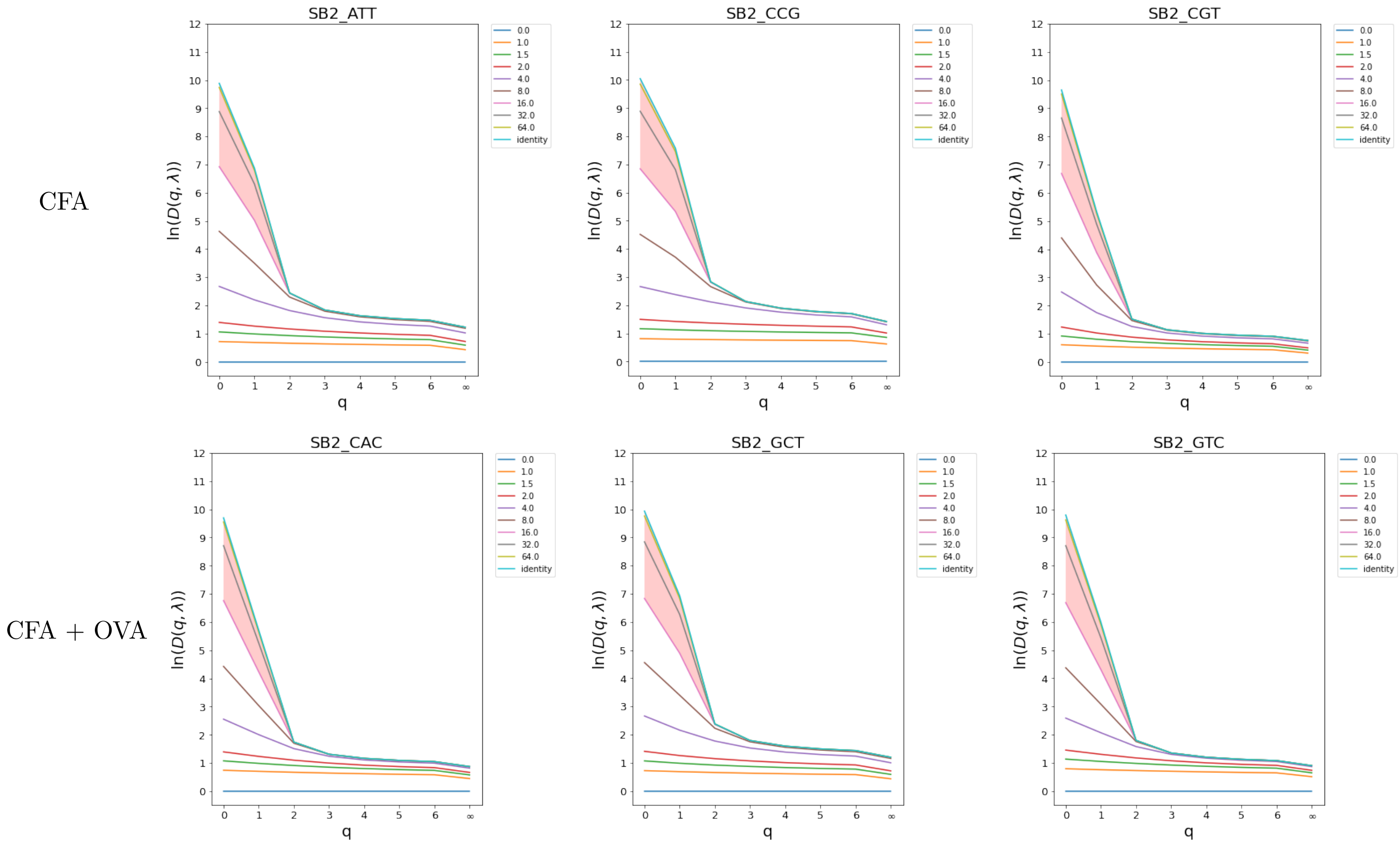

Supplementary Figure 14: Diversity profiles from repertoires of the immunised mice group in the murine dataset collected **60 days** post immunisation. CDR$_\beta$3 distance calculated using Atchley factor distance.

Supplementary Note 2.5: PCA on natural logarithm transformed values of diversity with Atchley factor distance as CDR3$_\beta$ distance

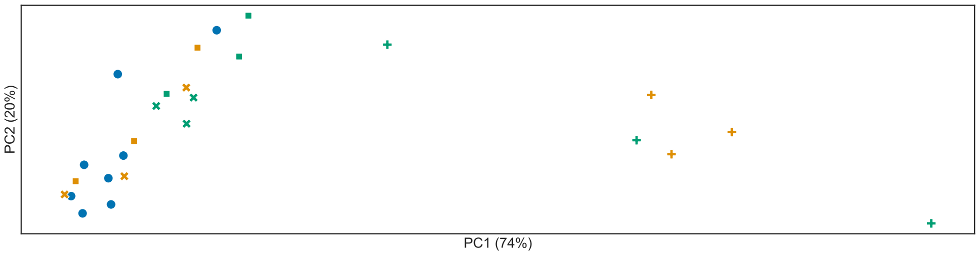

(a)

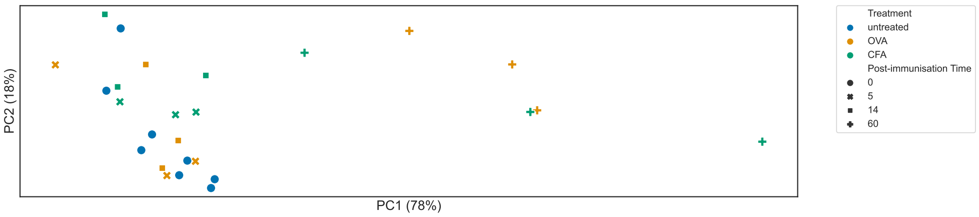

(b)

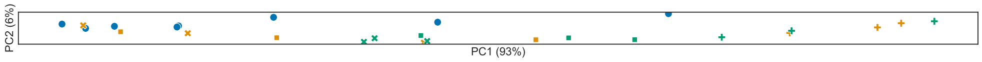

(c)

Supplementary Figure 15: Principal Components Analysis on diversity calculated for the murine dataset using the Atchley Factor distance for TCRs. The aspect ratio corresponds to variation found by PCA. **a.** PCA on features extracted from the diversity profiles constructed from the true diversity $D(q,\lambda)$. **b.** PCA on values of true diversity $D(q,\lambda)$. **c.** PCA on naive diversity values $D(q)$, i.e. $\lambda =$ identity.

Supplementary Note 2.6: DivP features relationships with Atchley factor distance as $CDR3_\beta$ distance

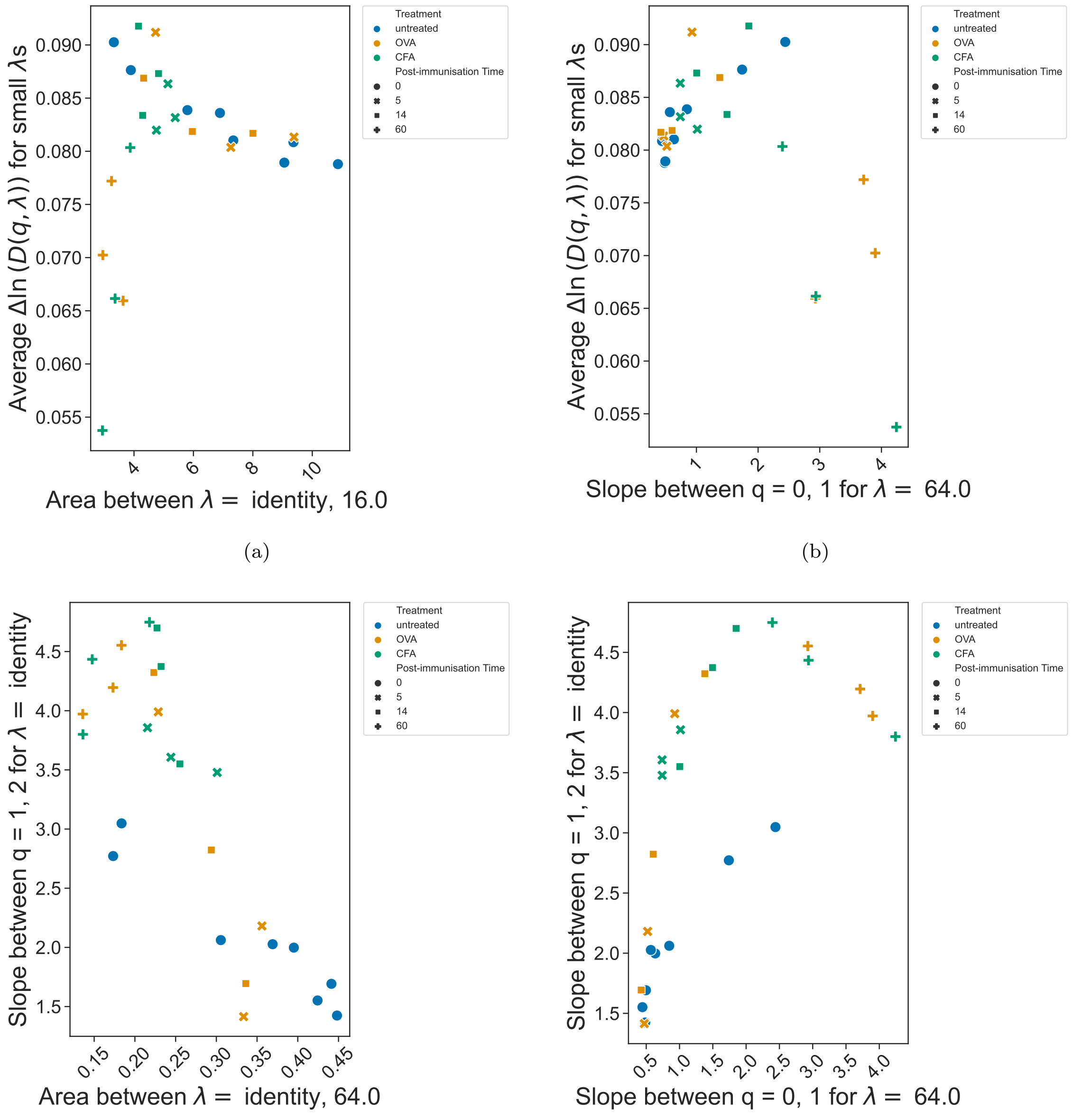

(a) (b) (c) (d)

Supplementary Figure 16: Graphs showing relationships between some of the divP features. **a.** average $\Delta \ln D(q, \lambda)$ for small λs is shown versus the area between curves of $\lambda =$ identity and 16.0; **b.** average $\Delta \ln D(q, \lambda)$ for small λs is shown versus the slope of $q = 0 \to 1$ for value of λ 64.0; **c.** slope of $q = 1 \to 2$ for value of λ identity (i.e. naive diversity) is shown versus the area between curves of $\lambda =$ identity and **d.** 64.0; slope of $q = 1 \to 2$ for value of λ identity (i.e. naive diversity) is shown versus the slope of $q = 0 \to 1$ for value of λ 64.0.

Supplementary Note 2.7: Trends of three features extracted from divPs versus timepoint and treatment regime with Atchley factor distance as CDR3$_\beta$ distance

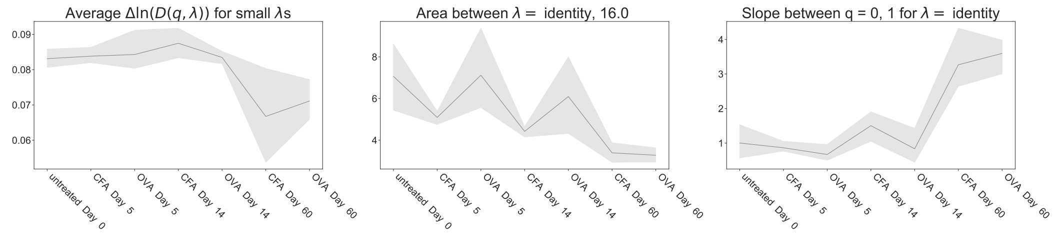

Supplementary Figure 17: Trends of three features extracted from divPs are shown versus the treatment regime and timepoints ending with the latest timepoint. The features are, from left to right: average $\Delta \ln D(q, \lambda)$ for small λs, between curves of $\lambda =$ identity and 16.0 and slope of $q = 0 \rightarrow 1$ for value of λ identity. The line connects the mean values of the features for all samples within a group and the shaded area represents the confidence interval.

Supplementary Note 2.8: Principal Components Analysis of diversity values from the randomised murine dataset with random frequencies with BLOSUM45 as CDR$_\beta$3 distance

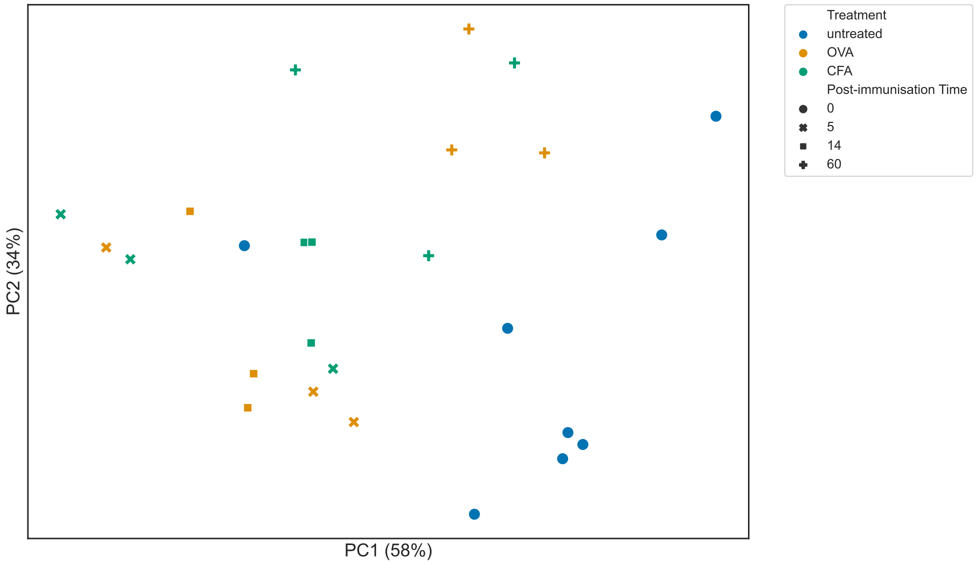

(a)

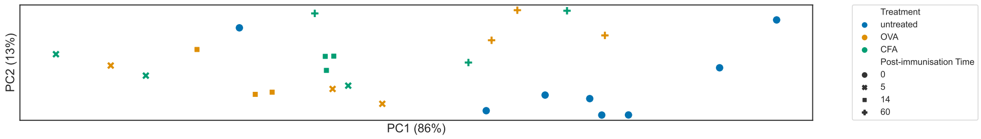

(b)

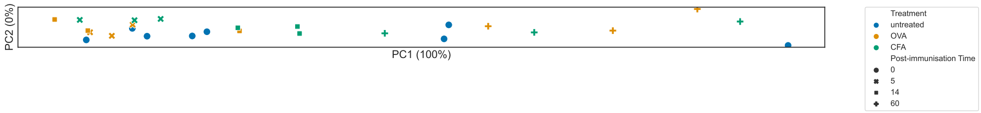

(c)

Supplementary Figure 18: Principal Components Analysis on diversity calculated for the randomised murine dataset. The aspect ratio corresponds to variation ound by PCA. **a.** PCA on features extracted from the diversity profiles constructed from the true diversity $D(q, \lambda)$. textbfb. PCA on values of true diversity $D(q, \lambda)$. **c.** PCA on naive diversity values $D(q)$, i.e. λ =identity.

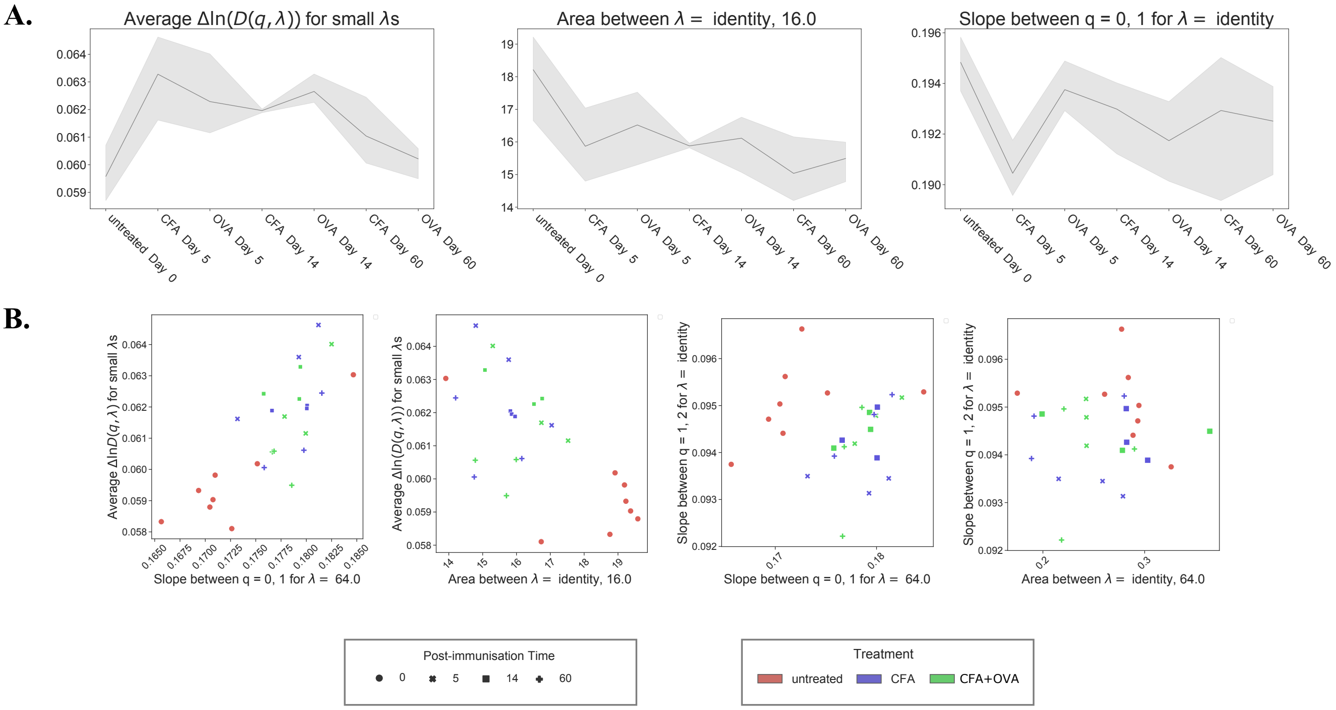

Supplementary Figure 19: **A.** Trends of three features for the randomised murine dataset are shown versus the treatment regime and timepoints ending with the latest timepoint. The features are, from left to right: average $\Delta \ln(D(q,\lambda))$ for small λs, between curves of $\lambda =$identity and 16.0 and slope of $q = 0 \rightarrow 1$ for value of $\lambda =$identity. The line connects the mean values of the features or all samples within a group and the shaded area represents the confidence interval. **B.** Graphs showing relationships between some f the divP features of the murine dataset with random frequencies. From left to right: average $\Delta \ln(D(q,\lambda))$ for small λs is shown versus the slope of $q = 0 \rightarrow 1$ for value of λ 64.0; average $\Delta \ln(D(q,\lambda))$ for small λs is shown versus the area between curves of $\lambda =$identity and 16.0; slope of $q = 1 \rightarrow 2$ for value of $\lambda =$identity (i.e. naive diversity) is shown versus the slope of $q = 0 \rightarrow 1$ for value of λ 64.0; slope of $q = 1 \rightarrow 2$ for value of $\lambda =$identity (i.e. naive diversity) is shown versus the area between curves of $\lambda =$identity and 64.0.

Supplementary Note 3: Human Dataset analysis

Supplementary Table 2: Human Dataset Subsampling: Overview of number CDR3 clones prior and post subsampling in bulk TCR repertoires of the human dataset.

Sample name	RECIST criteria	Sample collection time (days)	Number clones prior to sampling	Number of clones after sampling
Pt10_PD_PBMC_Day0	PD	0	261326	39555
Pt16_PD_PBMC_Day0	PD	0	170417	36110
Pt27_PD_PBMC_Day0	PD	0	102012	25848
Pt28_PD_PBMC_Day0	PD	0	197523	38151
Pt36_PD_PBMC_Day0	PD	0	42953	21211
Pt38_PD_PBMC_Day0	PD	0	69248	18942
Pt40_PD_PBMC_Day0	PD	0	119828	20905
Pt43_PD_PBMC_Day0	PD	0	144548	32998
Pt10_PD_PBMC_Day22	PD	22	261955	39417
Pt16_PD_PBMC_Day22	PD	22	182238	35294
Pt27_PD_PBMC_Day22	PD	22	30562	17699
Pt28_PD_PBMC_Day22	PD	22	162481	36018
Pt36_PD_PBMC_Day22	PD	22	52416	22467
Pt38_PD_PBMC_Day22	PD	22	83106	20022
Pt40_PD_PBMC_Day22	PD	22	153595	26737
Pt43_PD_PBMC_Day22	PD	22	149218	32983
Pt5_SD_PBMC_Day0	SD	0	124049	36074
Pt9_SD_PBMC_Day0	SD	0	56702	21371
Pt22_SD_PBMC_Day0	SD	0	72589	26421
Pt30_SD_PBMC_Day0	SD	0	74019	17423
Pt32_SD_PBMC_Day0	SD	0	75631	18287
Pt5_SD_PBMC_Day22	SD	22	150203	36579
Pt9_SD_PBMC_Day22	SD	22	43496	20669
Pt22_SD_PBMC_Day22	SD	22	97833	30146
Pt30_SD_PBMC_Day22	SD	22	82704	20291
Pt32_SD_PBMC_Day22	SD	22	59017	19849
Pt1_PR_PBMC_Day0	PR	0	174555	33446
Pt17_PR_PBMC_Day0	PR	0	169671	37698
Pt23_PR_PBMC_Day0	PR	0	91260	16753
Pt37_PR_PBMC_Day0	PR	0	130788	33824
Pt44_PR_PBMC_Day0	PR	0	108678	29786
Pt1_PR_PBMC_Day22	PR	22	205316	33568
Pt17_PR_PBMC_Day22	PR	22	229810	32628
Pt23_PR_PBMC_Day22	PR	22	94146	17549
Pt37_PR_PBMC_Day22	PR	22	195069	35062
Pt44_PR_PBMC_Day22	PR	22	120658	28538
Pt3_CR_PBMC_Day0	CR	0	128860	29075
Pt4_CR_PBMC_Day0	CR	0	119253	27209
Pt3_CR_PBMC_Day22	CR	22	92123	27116
Pt4_CR_PBMC_Day22	CR	22	108383	29732

Supplementary Note 3.1: Diversity profiles of the human dataset calculated using BLOSUM45 CDR$_{\beta}$3 distances

Diversity profiles calculated for the human dataset with 50000 subsample size. The profiles are organised according to RECIST criteria and timepoint in Tables 5 to 12 for progressive disease (PD) day 0 and 22, stable disease (SD) day 0 and 22, partial responders (PR) day 0 and 22 and complete responders (CR) day 0 and 22, respectively. The distance metric used in estimating diversity was based on the BLOSUM45 alignment.

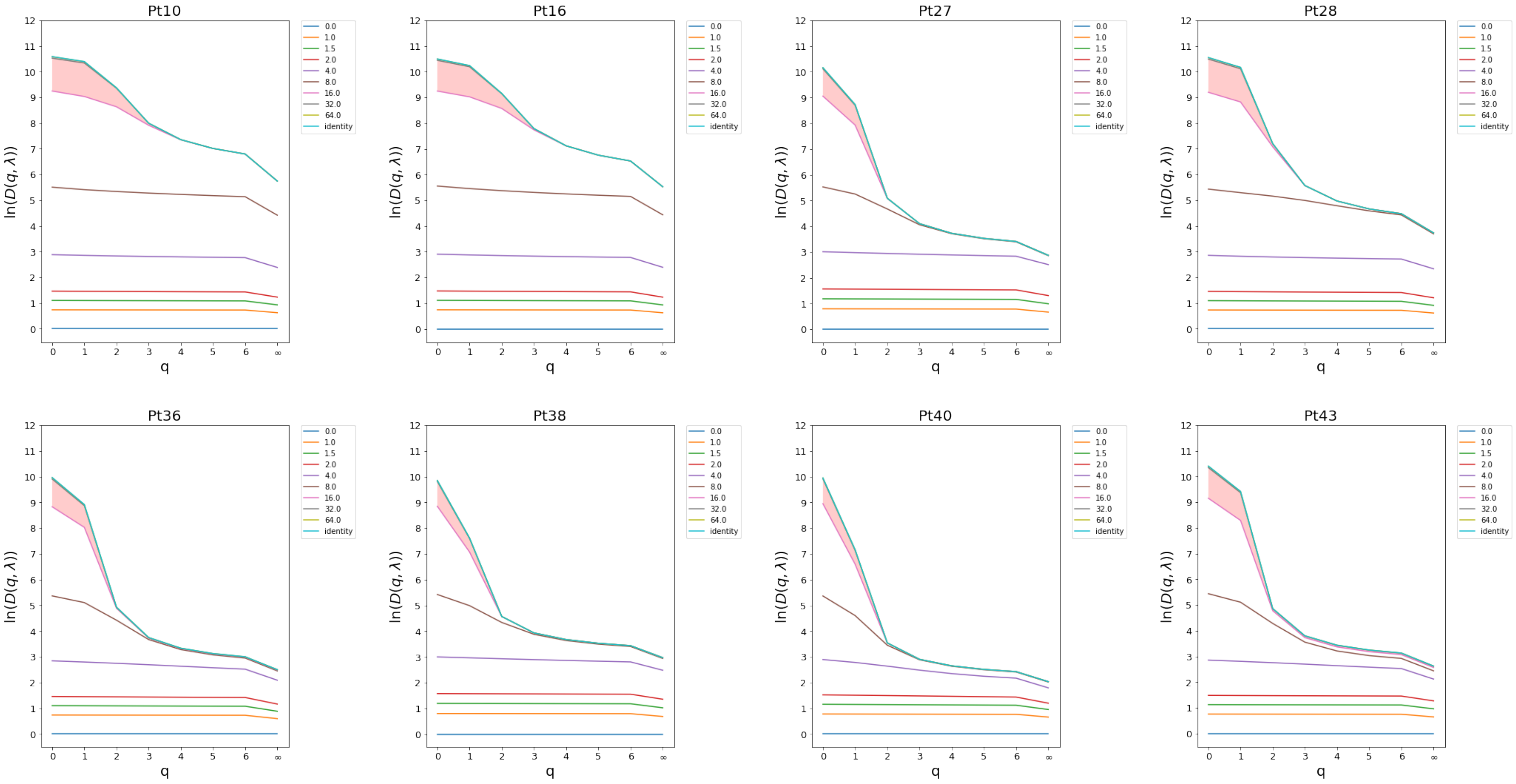

Supplementary Figure 20: Progressive Disease (PD) Day 0

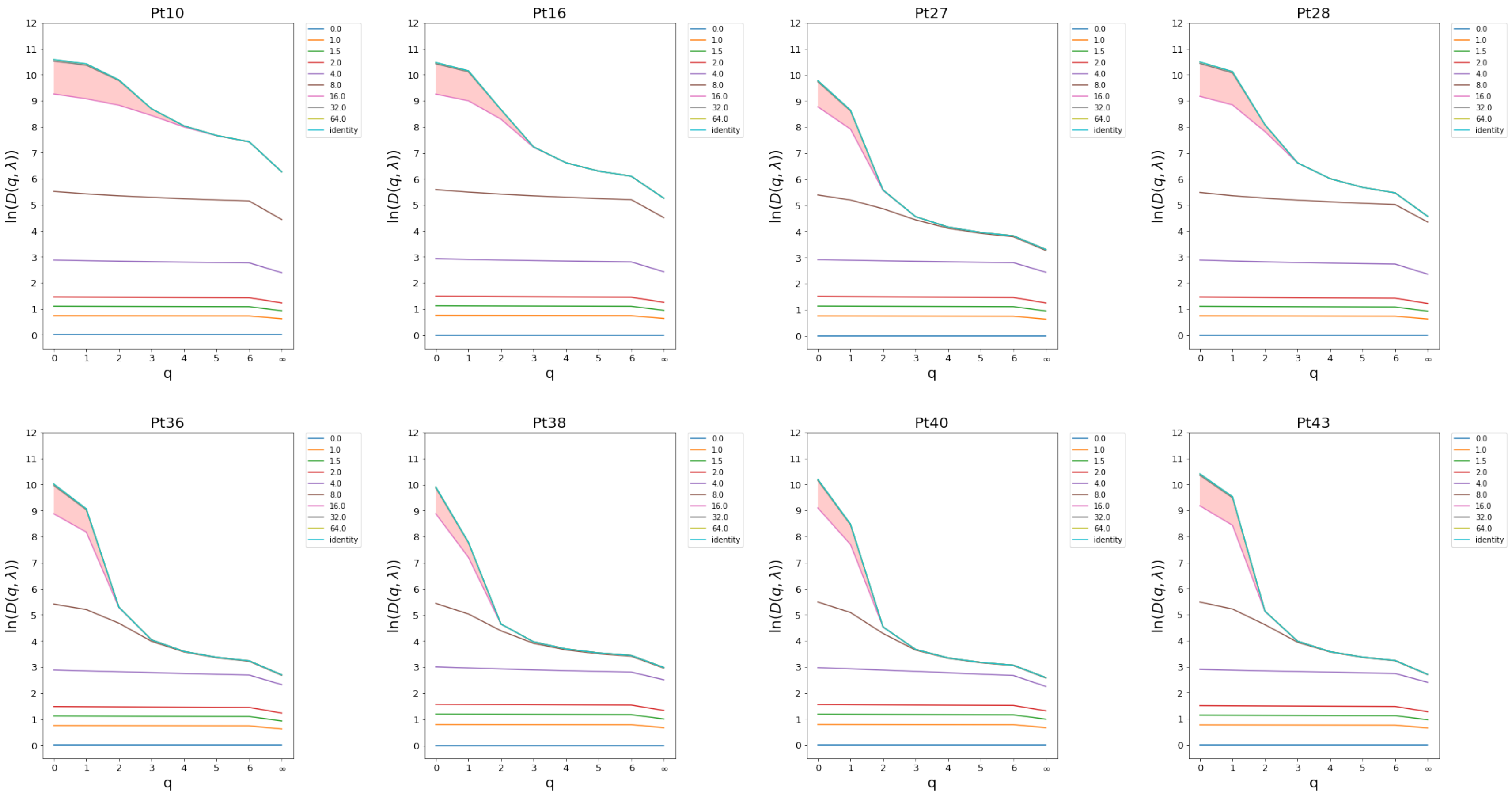

Supplementary Figure 21: Progressive Disease (PD) Day 22

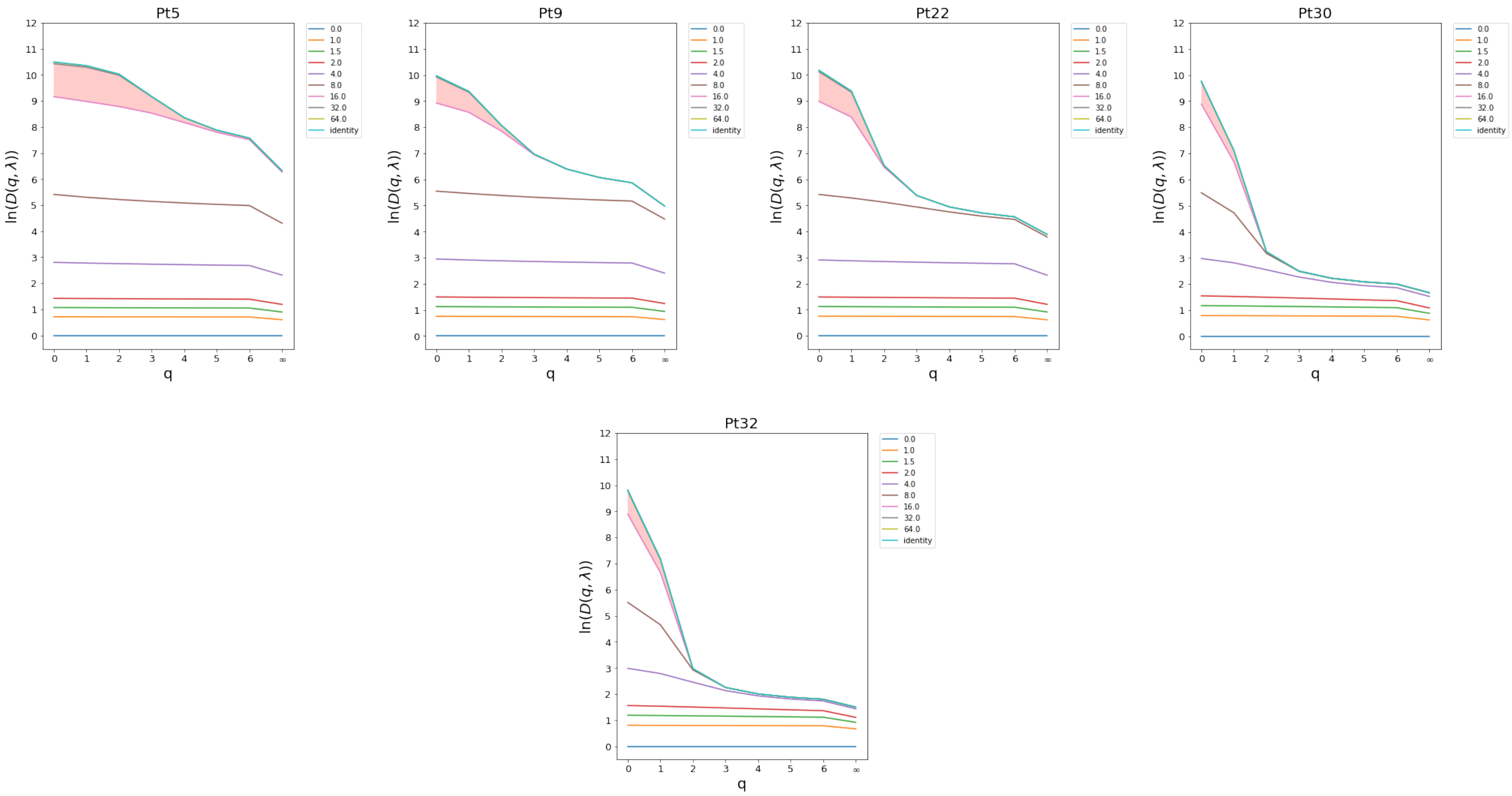

Supplementary Figure 22: Stable Disease (SD) Day 0

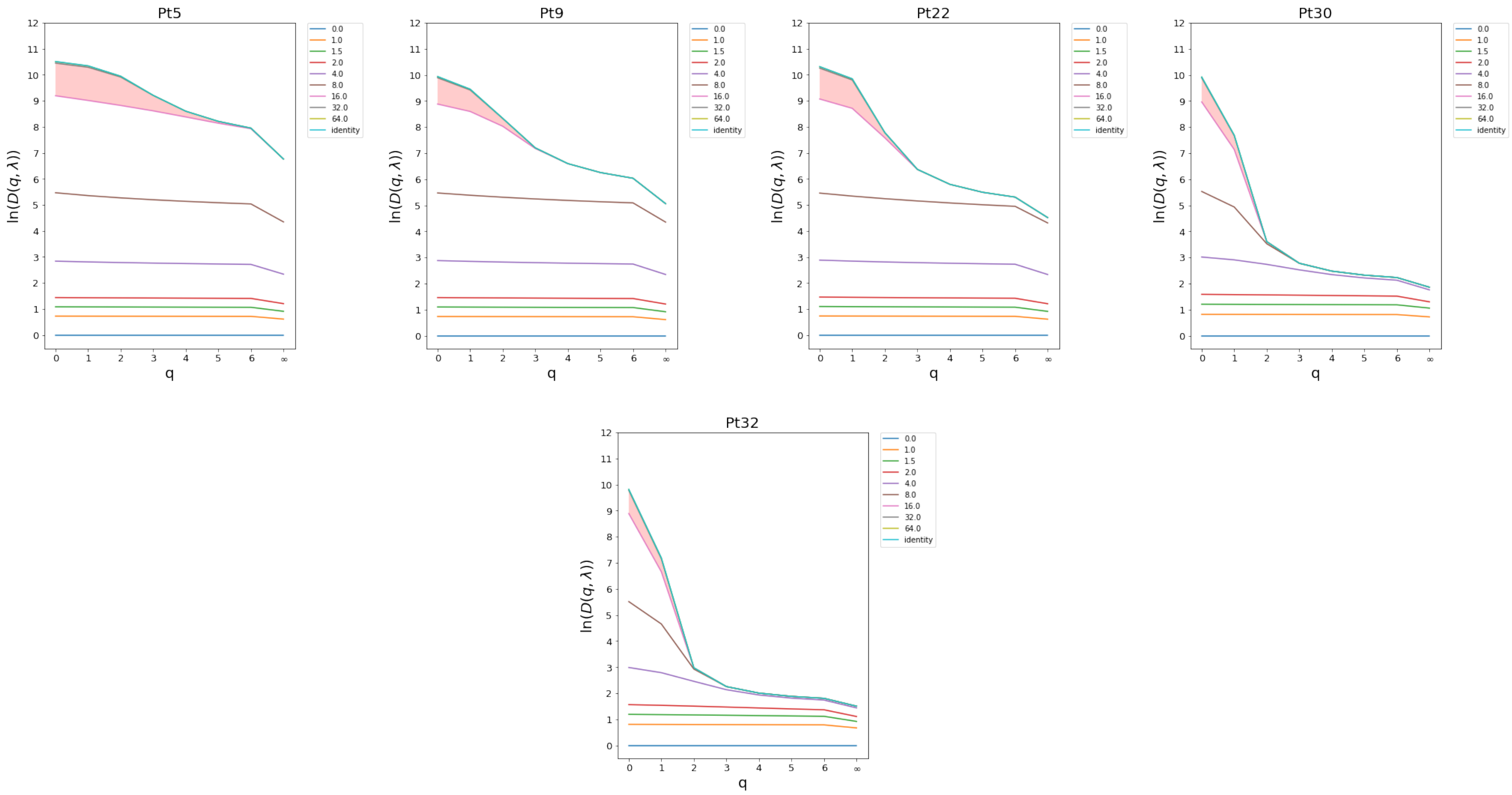

Supplementary Figure 23: Stable Disease (SD) Day 22

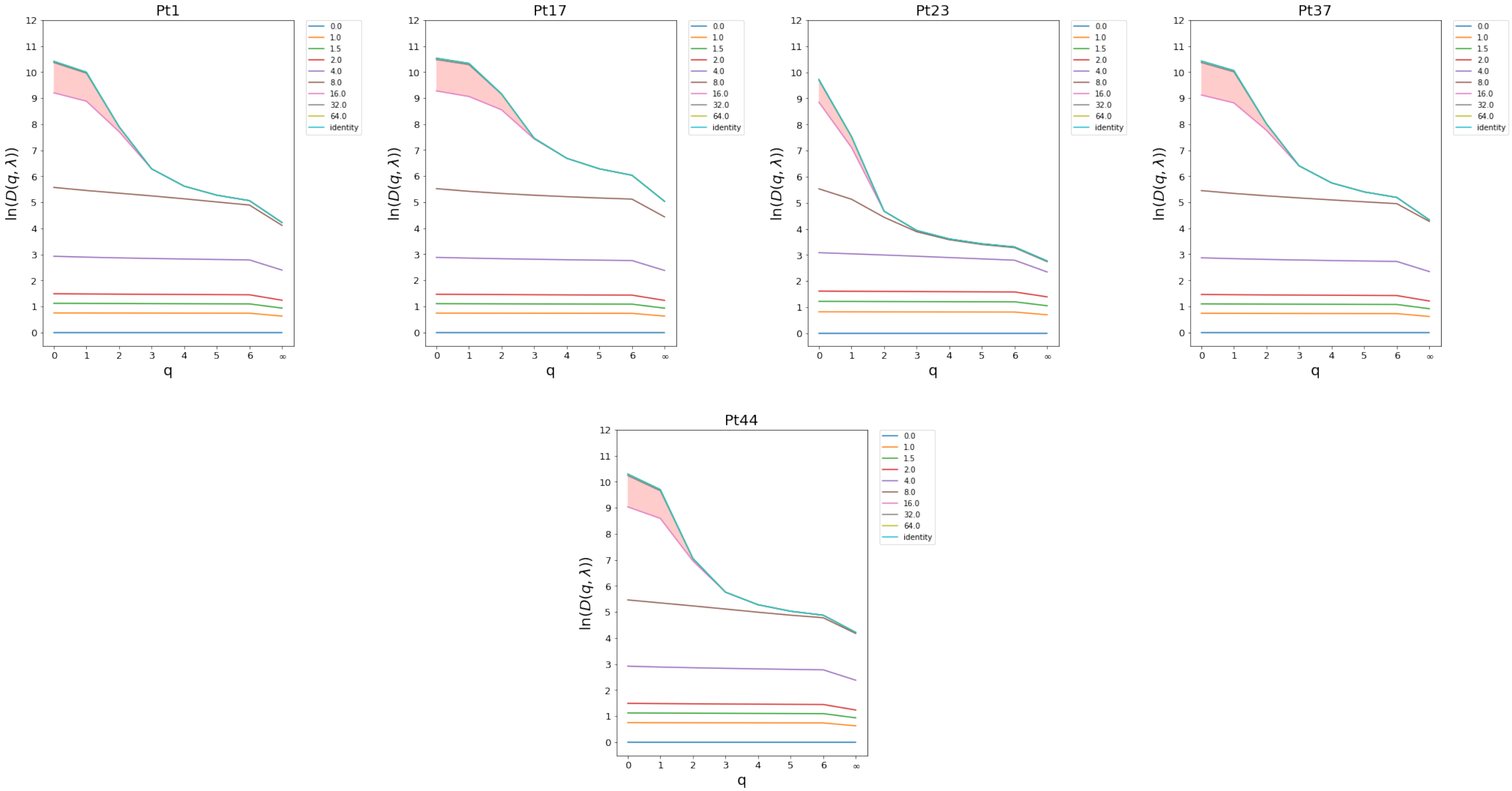

Supplementary Figure 24: Partial Responders (PR) Day 0

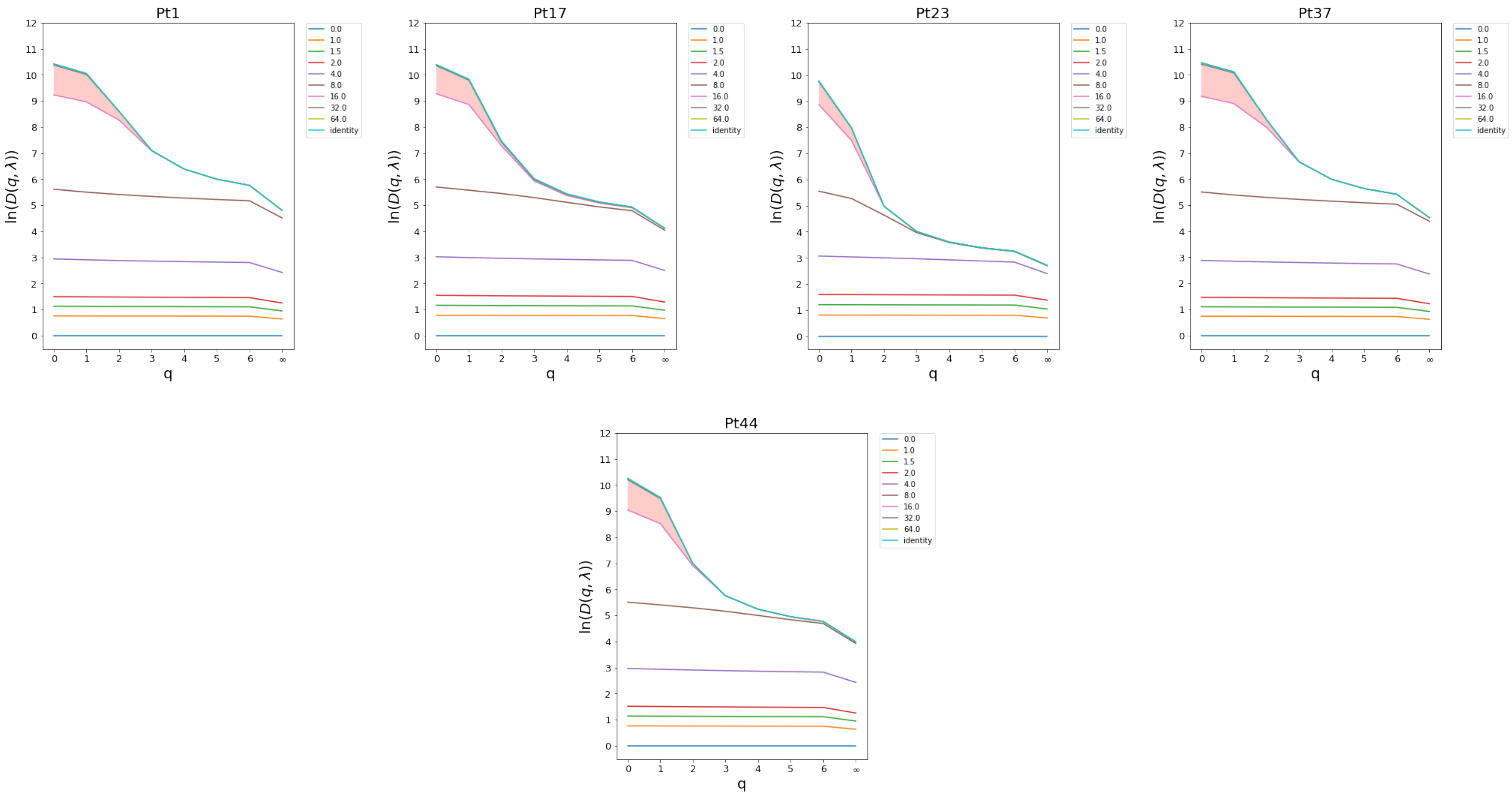

Supplementary Figure 25: Partial Responders (PR) Day 22

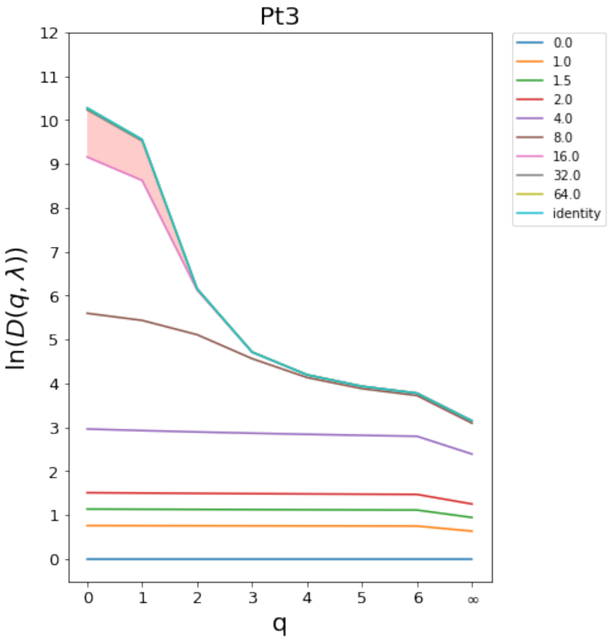

Supplementary Figure 26: Complete Responders (CR) Day 0

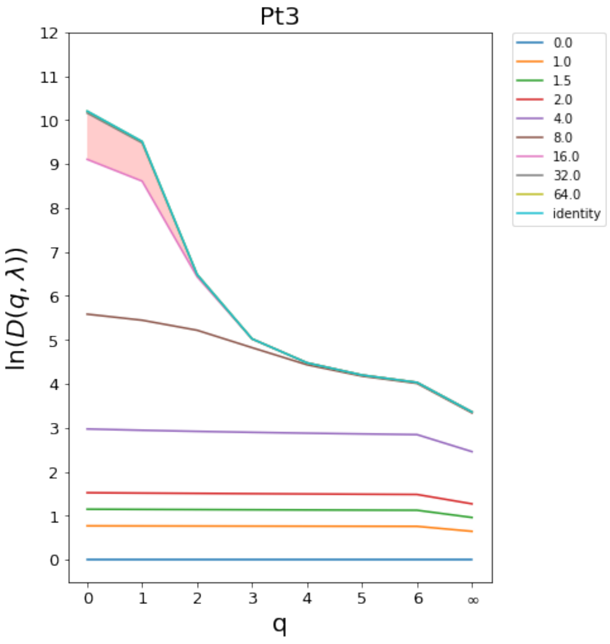

Supplementary Figure 27: Complete Responders (CR) Day 22

Supplementary Note 3.2: Principal Components Analysis of diversity values from the human dataset with BLOSUM45 as CDR$_\beta$3 distance

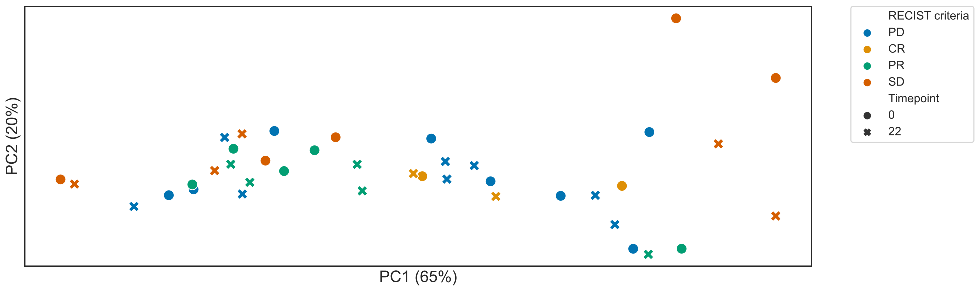

(a)

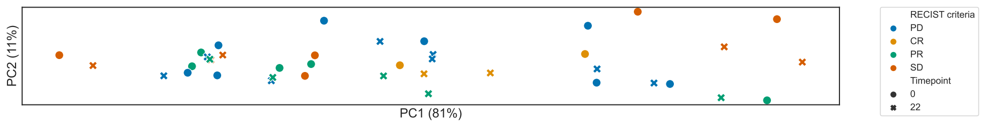

(b)

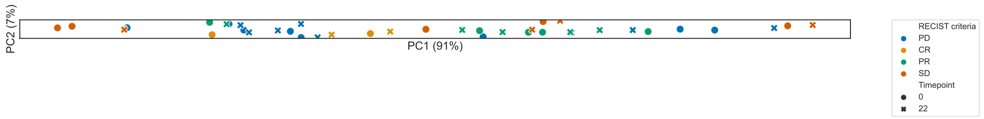

(c)

Supplementary Figure 28: Principal Components Analysis on diversity calculated for the human dataset. The aspect ratio corresponds to variation found by PCA. **a.** PCA on features extracted from the diversity profiles constructed from the true diversity $D(q, \lambda)$. **b.** PCA on values of true diversity $D(q, \lambda)$. **c.** PCA on naive diversity values $D(q)$, i.e. $\lambda =$ identity.

Supplementary Note 3.3: DivP features relationships from the human dataset with BLOSUM45 distance as CDR3$_\beta$ distance

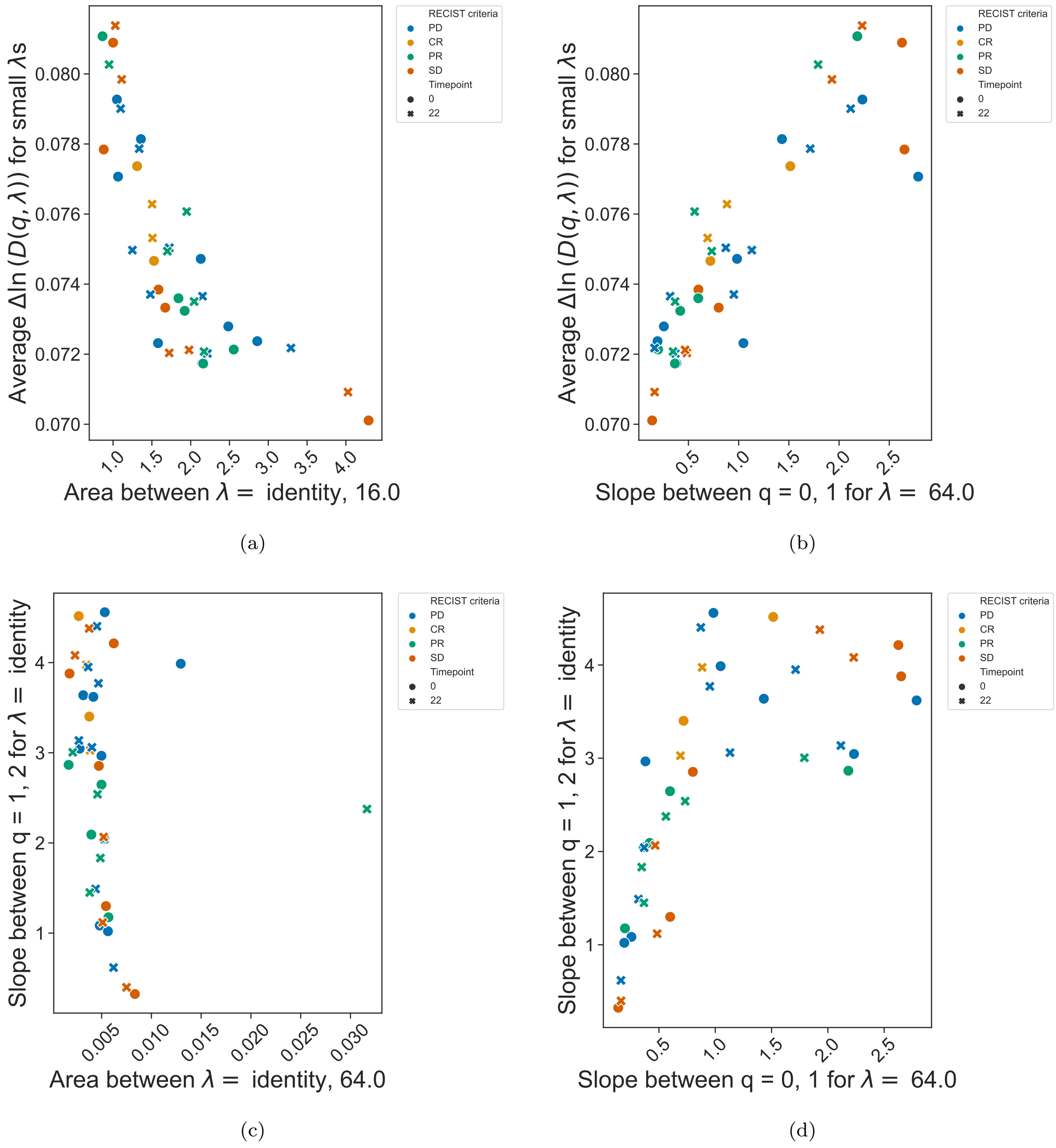

Supplementary Figure 29: Graphs showing relationships between some of the divP features. **a.** average $\Delta \ln D(q, \lambda)$ for small λs is shown versus the area between curves of λ = identity and 16.0; **b.** average $\Delta \ln D(q, \lambda)$ for small λs is shown versus the slope of $q = 0 \to 1$ for value of λ 64.0; **c.** slope of $q = 1 \to 2$ for value of λ identity (i.e. naive diversity) is shown versus the area between curves of λ = identity and **d.** 64.0; slope of $q = 1 \to 2$ for value of λ identity (i.e. naive diversity) is shown versus the slope of $q = 0 \to 1$ for value of λ 64.0.

Supplementary Note 3.4: Principal Components Analysis of diversity values from the randomised human dataset with random frequencies with BLOSUM45 as CDR$_\beta$3 distance

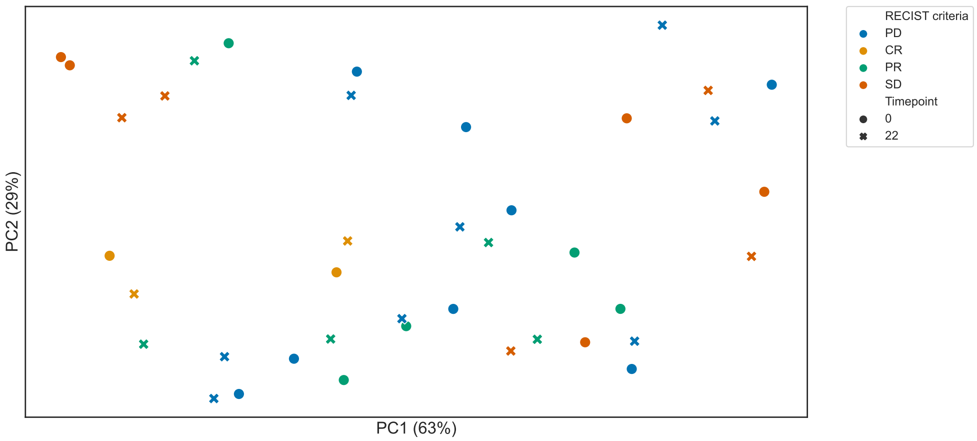

(a)

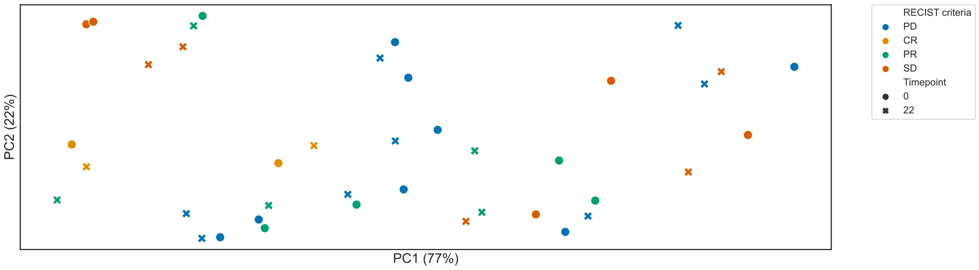

(b)

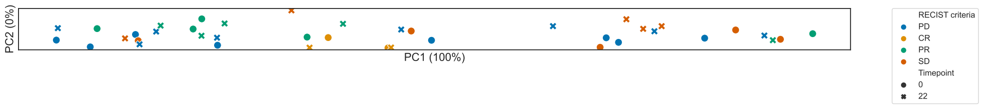

(c)

Supplementary Figure 30: Principal Components Analysis on diversity calculated for the randomised human dataset. **a.** PCA on features extracted from the diversity profiles constructed from the true diversity $D(q, \lambda)$. **b.** PCA on values of true diversity $D(q, \lambda)$. **c.** PCA on naive diversity values $D(q)$, i.e. $\lambda =$ identity.

Supplementary Note 3.5: Features of diversity profiles from the randomised human dataset with random frequencies with BLOSUM45 as $\mathrm{CDR}_\beta 3$ distance

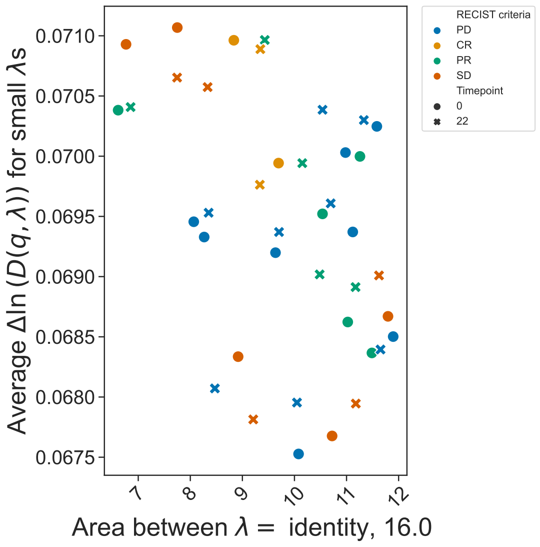

(a)

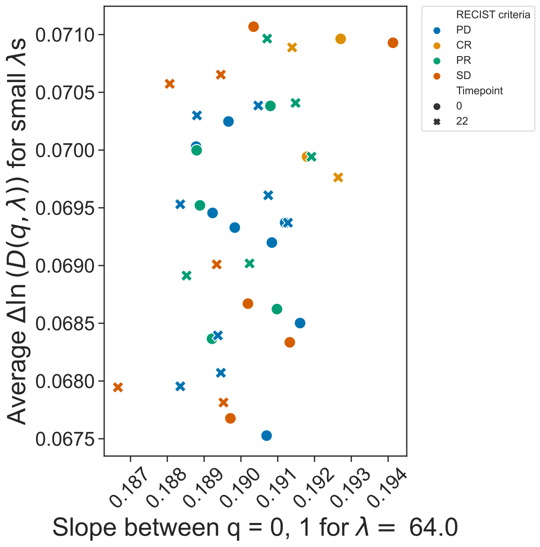

(b)

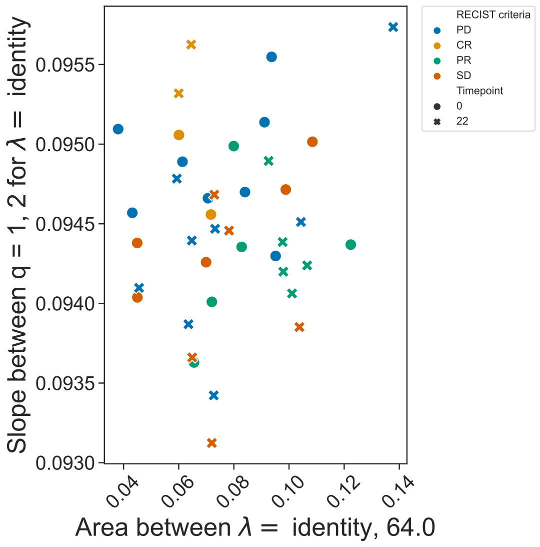

(c)

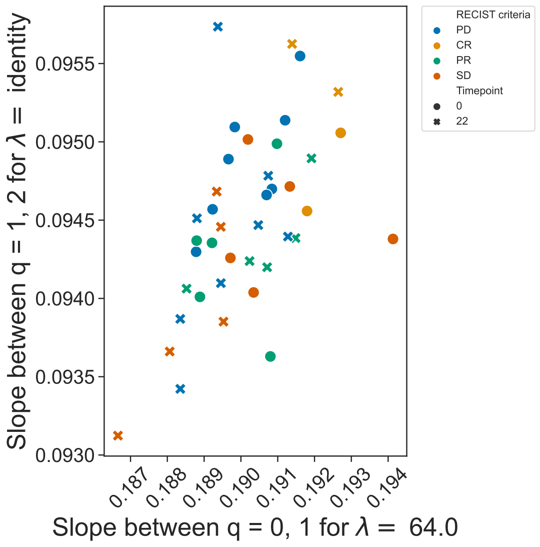

(d)

Supplementary Figure 31: Graphs showing relationships between some of the divP features of the human randomised dataset. **a.** average $\Delta \ln D(q,\lambda)$ for small λs is shown versus the area between curves of $\lambda =$ identity and 16.0; **b.** average $\Delta \ln D(q,\lambda)$ for small λs is shown versus the slope of $q = 0 \rightarrow 1$ for value of λ 64.0; **c.** slope of $q = 1 \rightarrow 2$ for value of λ identity (i.e. naive diversity) is shown versus the area between curves of $\lambda =$ identity and **d.** 64.0; slope of $q = 1 \rightarrow 2$ for value of λ identity (i.e. naive diversity) is shown versus the slope of $q = 0 \rightarrow 1$ for value of λ 64.0.
