# Supplementary material for: Signatures of T cell immunity revealed using sequence similarity with TCRDivER algorithm
Source: Commun Biol. 2023 Mar 31;6:357. doi: 10.1038/s42003-023-04702-8 (PMC10066310; doi:10.1038/s42003-023-04702-8)
Supplement: Supplementary file 3 — Reporting Summary [file 42003_2023_4702_MOESM3_ESM.pdf]

nature portfolio

Corresponding author(s): Milena Vujović
Last updated by author(s): Jan 8, 2023

Reporting Summary

Nature Portfolio wishes to improve the reproducibility of the work that we publish. This form provides structure for consistency and transparency in reporting. For further information on Nature Portfolio policies, see our Editorial Policies and the Editorial Policy Checklist.

Statistics

For all statistical analyses, confirm that the following items are present in the figure legend, table legend, main text, or Methods section.

n/a	Confirmed	
☐	☒	The exact sample size (*n*) for each experimental group/condition, given as a discrete number and unit of measurement
☐	☒	A statement on whether measurements were taken from distinct samples or whether the same sample was measured repeatedly
☐	☒	The statistical test(s) used AND whether they are one- or two-sided<br>*Only common tests should be described solely by name; describe more complex techniques in the Methods section.*
☐	☒	A description of all covariates tested
☐	☒	A description of any assumptions or corrections, such as tests of normality and adjustment for multiple comparisons
☐	☒	A full description of the statistical parameters including central tendency (e.g. means) or other basic estimates (e.g. regression coefficient) AND variation (e.g. standard deviation) or associated estimates of uncertainty (e.g. confidence intervals)
☐	☒	For null hypothesis testing, the test statistic (e.g. *F*, *t*, *r*) with confidence intervals, effect sizes, degrees of freedom and *P* value noted<br>*Give P values as exact values whenever suitable.*
☒	☐	For Bayesian analysis, information on the choice of priors and Markov chain Monte Carlo settings
☐	☒	For hierarchical and complex designs, identification of the appropriate level for tests and full reporting of outcomes
☐	☒	Estimates of effect sizes (e.g. Cohen's *d*, Pearson's *r*), indicating how they were calculated

Our web collection on statistics for biologists contains articles on many of the points above.

Software and code

Policy information about availability of computer code

Data collection	Data was collected from public repositories from previously published studies, as is described in more detail in the manuscript.
Data analysis	All the custom computer code used to analyse data in this study can be found at: https://github.com/sciencisto/TCRDivER. The code has been written by MV in Python. A detailed description of the code is included in the manuscript.

For manuscripts utilizing custom algorithms or software that are central to the research but not yet described in published literature, software must be made available to editors and reviewers. We strongly encourage code deposition in a community repository (e.g. GitHub). See the Nature Portfolio guidelines for submitting code & software for further information.

Data

Policy information about availability of data

All manuscripts must include a data availability statement. This statement should provide the following information, where applicable:

- Accession codes, unique identifiers, or web links for publicly available datasets
- A description of any restrictions on data availability
- For clinical datasets or third party data, please ensure that the statement adheres to our policy

The datasets generated during and/or analysed during the current study are available in the ncbi repository, http://www.ncbi.nlm.nih.gov/sra/?term=SRP075893 and the Adaptive Bioctechnologies ImmunoSEQ database, https://doi.org/10.21417/B7BW6X.

Human research participants

Policy information about studies involving human research participants and Sex and Gender in Research.

Reporting on sex and gender	Not applicable as we have not analysed only data from public databases The only information available was sex. Any other considerations on gender were not available to us.
Population characteristics	Not applicable as we have analysed only data from public databases.
Recruitment	Not applicable as we have analysed only data from public databases.
Ethics oversight	Not applicable as we have analysed only data from public databases.

Note that full information on the approval of the study protocol must also be provided in the manuscript.

Field-specific reporting

Please select the one below that is the best fit for your research. If you are not sure, read the appropriate sections before making your selection.

☒ Life sciences ☐ Behavioural & social sciences ☐ Ecological, evolutionary & environmental sciences

For a reference copy of the document with all sections, see nature.com/documents/nr-reporting-summary-flat.pdf

Life sciences study design

All studies must disclose on these points even when the disclosure is negative.

Sample size	There was no experimental work involved in this study. All data was collected from publicly available repositories.
Data exclusions	There was no experimental work involved in this study. All data was collected from publicly available repositories. Repertoire subsampling was performed computationally to accommodate for computational speed. Further advancements in computational power will lead to larger subsampling sizes.
Replication	There was no experimental work involved in this study. All data was collected from publicly available repositories.
Randomization	There was no experimental work involved in this study. All data was collected from publicly available repositories.
Blinding	There was no experimental work involved in this study. All data was collected from publicly available repositories.

Reporting for specific materials, systems and methods

We require information from authors about some types of materials, experimental systems and methods used in many studies. Here, indicate whether each material, system or method listed is relevant to your study. If you are not sure if a list item applies to your research, read the appropriate section before selecting a response.

Materials & experimental systems

n/a	Involved in the study	
☒	☐	Antibodies
☒	☐	Eukaryotic cell lines
☒	☐	Palaeontology and archaeology
☒	☐	Animals and other organisms
☒	☐	Clinical data
☒	☐	Dual use research of concern

Methods

n/a	Involved in the study	
☒	☐	ChIP-seq
☒	☐	Flow cytometry
☒	☐	MRI-based neuroimaging

March 2021
